# Prophylactic versus Therapeutic Fingolimod: Restoration of Presynaptic Defects in Mice Suffering from Experimental Autoimmune Encephalomyelitis

**DOI:** 10.1371/journal.pone.0170825

**Published:** 2017-01-26

**Authors:** Tommaso Bonfiglio, Guendalina Olivero, Elisa Merega, Silvia Di Prisco, Cristina Padolecchia, Massimo Grilli, Marco Milanese, Lorenzo Di Cesare Mannelli, Carla Ghelardini, Giambattista Bonanno, Mario Marchi, Anna Pittaluga

**Affiliations:** 1 Department of Pharmacy, Pharmacology and Toxicology Section, School of Medical and Pharmaceutical Sciences, University of Genoa, Genoa, Italy; 2 Center of Excellence for Biomedical Research, University of Genoa, Genoa, Italy; 3 Department of Neuroscience, Psychology, Drug Research and Child Health, Neurofarba, Pharmacology and Toxicology section, University of Florence, Florence, Italy; Uniformed Services University of the Health Sciences F Edward Hebert School of Medicine, UNITED STATES

## Abstract

Fingolimod, the first oral, disease-modifying therapy for MS, has been recently proposed to modulate glutamate transmission in the central nervous system (CNS) of mice suffering from Experimental Autoimmune Encephalomyelitis (EAE) and in MS patients. Our study aims at investigating whether oral fingolimod recovers presynaptic defects that occur at different stages of disease in the CNS of EAE mice. *In vivo* prophylactic (0.3 mg/kg for 14 days, from the 7^th^ day post immunization, d.p.i, the drug dissolved in the drinking water) fingolimod significantly reduced the clinical symptoms and the anxiety-related behaviour in EAE mice. Spinal cord inflammation, demyelination and glial cell activation are markers of EAE progression. These signs were ameliorated following oral fingolimod administration. Glutamate exocytosis was shown to be impaired in cortical and spinal cord terminals isolated from EAE mice at 21 ± 1 d.p.i., while GABA alteration emerged only at the spinal cord level. Prophylactic fingolimod recovered these presynaptic defects, restoring altered glutamate and GABA release efficiency. The beneficial effect occurred in a dose-dependent, region-specific manner, since lower (0.1–0.03 mg/kg) doses restored, although to a different extent, synaptic defects in cortical but not spinal cord terminals. A delayed reduction of glutamate, but not of GABA, exocytosis was observed in hippocampal terminals of EAE mice at 35 d.p.i. Therapeutic (0.3 mg/kg, from 21 d.p.i. for 14 days) fingolimod restored glutamate exocytosis in the cortex and in the hippocampus of EAE mice at 35 ± 1 d.p.i. but not in the spinal cord, where also GABAergic defects remained unmodified. These results improve our knowledge of the molecular events accounting for the beneficial effects elicited by fingolimod in demyelinating disorders.

## Introduction

Multiple sclerosis (MS) is mediated by an immune attack directed at myelin, which leads to a progressively degenerating disorder of the central nervous system (CNS). Although immunological mechanisms are responsible for the majority of the cascade of events leading to MS, pathogenetic events involving neurons and astrocytes have been recently implicated in the pathogenesis of this disease [1–5].

More precisely, recent work has targeted glutamate and GABA transmission at chemical synapsis in the CNS of EAE mice and MS patients. Central glutamatergic and GABAergic neuronal defects were proposed to determine synaptic pathology in MS patients and in animals affected by Experimental Autoimmune Encephalomyelitis (EAE). These impairments might account for a reduced ability of CNS to cope with central neuro-injuries. In particular, the endogenous bioavailability of glutamate in the cerebrospinal fluid of MS patients as well as EAE mice was altered with respect to healthy individuals [6]. Glutamate release efficiency was augmented in the striatum and the spinal cord [4,7–11], but was significantly reduced in cortical nerve endings of EAE animals [8,9,12,13]. A decrease in the inhibitory amino acid GABA and its synthesising enzyme GABA decarboxylase was also measured in the spinal cord of EAE guinea pigs and cerebrospinal fluid of MS patients [14,15] and a loss of inhibitory interneurons was detected in the brain of EAE mice [16].

Fingolimod (FTY720, Gilenya^®^) was the first oral, disease-modifying therapy for MS [17]. It reduces relapses, disability progression, and brain atrophy in patients suffering from the relapsing-remitting form of MS [18]. Fingolimod is a pro-drug that is rapidly phosphorylated to the active compound fingolimod-phosphate. By binding at the sphingosine-1-phosphate receptors (S1PR), the drug prevents the egress of lymphocytes and exerts central effects including neuroprotection and remyelination [19,20]. Besides its immunomodulatory activity, fingolimod is beneficial to central glutamate transmission in the CNS of EAE mice [21]. Inasmuch, it restores glutamate-mediated intercortical excitability in MS patients suffering from the relapsing-remitting form of disease [22]. In an attempt to provide further evidence supporting the beneficial effect of fingolimod in CNS, we investigated whether the continuous *in vivo* administration of this drug could ameliorate glutamate and GABA presynaptic abnormalities in selected CNS regions of EAE mice. Fingolimod was dissolved in the mice drinking water for 14 days, starting either from an early asymptomatic stage of the disease (prophylactic administration), or after the acute stage of disease (therapeutic administration). Depending on the posology adopted, fingolimod ameliorated in a region-dependent manner glutamate and GABA release efficiency in the CNS of EAE mice. Both prophylactic and therapeutic administration restored glutamate release efficiency in the cortex and in the hippocampus, while prophylactic, but not therapeutic, fingolimod was beneficial at the spinal cord level.

## Materials and Methods

### Animals

Mice (female, strain C57BL/6J) were obtained from Charles River (Calco, Italy) and were housed in the animal facility of the Department of Pharmacy, Section of Pharmacology and Toxicology, School of Medical and Pharmaceutical Sciences, University of Genoa (authorization n. 484 of 2004, June, 8^th^). The experimental procedures were in strict accordance with the European legislation (Directive 2010/63/EU for animal experiments), with the ARRIVE guidelines, and they were approved by the Committee on the Ethics of Animal Experiments of the University of Genoa and by the Italian Ministry of Health (DDL 26/2014 and previous legislation; permit number 50/2011-B and number 612/2015-PR). All efforts were made to minimize animal suffering and the number of animals necessary to produce reliable results.

### EAE induction and clinical score

For EAE induction, mice (female, strain C57BL/6J, 18–20 g, 6–8 weeks) were immunized accordingly to a standard protocol [23], with minor modifications. Briefly, animals were subcutaneously injected with incomplete Freund’s adjuvant containing 4 mg/ml *Mycobacterium tuberculosis* (strain H37Ra) and 200 μg of the MOG_35–55_ peptide. Immunization with MOG_35–55_ was followed by i.p. administration of 250 ng of Pertussis toxin on day 0 and after 48 h. Clinical scores (0 = healthy; 1 = limp tail; 2 = ataxia and/or paresis of hindlimbs; 3 = paralysis of hindlimbs and/or paresis of forelimbs; 4 = tetraparalysis; 5 = moribund or death) were recorded daily. MOG_35-55_(+) EAE mice were killed by decapitation at 21 ± 1 or 35 ± 1 days post immunization (d.p.i.) as indicated. Control, non-immunized mice received the same treatment in the absence of the myelin antigen [MOG_35-55_(-) mice].

### Drug treatments

Mice were randomly assigned to the following groups: control, EAE, fingolimod-treated control, and fingolimod-treated EAE mice. Fingolimod, supplied by Novartis Pharma AG, was given orally, dissolved in the drinking water (concentration as indicated in the text). Recent studies demonstrated that the administration of fingolimod to EAE mice in the drinking water results in beneficial responses that are comparable to those observed following intraperitoneal injection ([24] and references therein) or gavage [25]. This route of administration was therefore adopted in order to minimize the daily handling and stress associated with the other methods of drug delivery. After a three-day trial to determine the amount of water consumed by each group of mice, mice were treated with fingolimod. The amount of drug dissolved in the water was adjusted daily to assure the correct dosage. When studying the effect of the prophylactic administration of fingolimod, the drug was added at the drinking water starting from 7 days post immunization (d.p.i.); that represents an asymptomatic stage of disease, for 14 days, till 21 d.p.i. We refer to this treatment as the “prophylactic fingolimod” treatment. When studying the effect of the administration of fingolimod starting from the acute stage of disease, the drug was added at the drinking water starting from 21 d.p.i.; that represents the stage of disease characterized by the most severe clinical symptoms. The drug was administered for 14 days, till 35 d.p.i. We refer to this treatment as the “therapeutic fingolimod” treatment. Either prophylactic or therapeutic fingolimod administration did not modify *per se* the daily water intake. The incidence of disease in the EAE mice enrolled in the study concerning therapeutic fingolimod was 100%, as only sick animals were included in the fingolimod untreated and treated EAE mice groups. As to the prophylactic treatment, drug administration started before the onset of the clinical symptoms, such that the disease incidence in these groups could not be predicted. However, in these studies, the untreated control EAE mice showed mild (*i*.*e*., mild to severe tail weakness, clinical score = 1.5) to serious (ataxia and/or paresis of hindlimbs, clinical score of about 2–2.5) clinical symptoms. Sick animals were not included in the study. Since the functional scores are not normally distributed data, the non parametric Wilcoxon analysis was applied to test the effect of the fingolimod treatment on functional recovery in EAE mice.

### Behavioural studies

#### Light dark box test

The light-dark box consists of a lighted and dark compartments (each comprising 35 cm x 30 cm x 21 cm). Animals were placed in the centre of the light zone, and they were allowed to explore the box for 10 min. Anxiety was analyzed in the light-dark box, by monitoring the time spent in the lighted compartment as well as by quantifying the number of crossings from the lighted to the dark side of the field [9,26].

#### Open field test

The open field consists of a square arena (34 x 34 cm with 24 cm walls) with walls made of translucent plastic. White lines were drawn on the floor of the box and divided into 9 squares. Mice were placed in the middle of the open field and were allowed to explore the field freely for 6 min. Thigmotaxis (the tendency to stay on the periphery of the open field) was quantified as time spent in the periphery (seconds, sec.) to evaluate anxiety-related behaviour [27].

### Histological analysis

#### Luxol Fast Blue

Twenty-one days after EAE induction, mice were sacrificed and the spinal cord (the lumbar part) was extracted. Formalin-fixed paraffin sections (7 μm) were prepared. Following standard dewaxing and rehydration, tissue sections were immersed overnight in 0.1% Luxol Fast Blue solution) at 56–60°C. Sections were rinsed in deionized water. Differentiation was initiated with immersion in 0.05% aqueous lithium carbonate for 15 sec followed by differentiation in multiple immersions in fresh 70% ethanol until grey and white matter could be distinguished, and nuclei decolorized. After washing in deionized water, sections were immersed in 0.8% Periodic Acid for 10 min and then rinsed in distilled water. Sections were incubated with Schiff ‘s reagent for 20 min and rinsed in distilled water for 15 min, and then dehydrated in 50 and 100% ethanol and coverslipped. Sections were examined using an Olympus BX40 microscope (Olympus, Milan, Italy) and photographed using a digital camera Olympus DP50 (Olympus, Milan, Italy).

#### Hematoxylin/Eosin

Hematoxylin has a deep blue-purple colour and stains nucleic acids. Eosin is pink and stains proteins non-specifically. In particular, nuclei are stained blue, whereas the cytoplasm and extracellular matrix have varying degrees of pink staining. Twenty-one days after EAE induction, mice were sacrificed and spinal cord was extracted. Formalin-fixed paraffin sections (7 μm) were collected. Following standard dewaxing and rehydration, tissue sections were immersed in eosin solution (Bio-optica, Milan, Italy) for 3 min. After 5–6 min running water, sections were immersed in hematoxylin solution (Bio-optica, Milan, Italy) for 3 min. After 5–6 min running water, sections were dehydrated and coverslipped. Sections were examined using an Olympus BX40 microscope (Olympus, Milan, Italy) and photographed using a digital camera Olympus DP50 (Olympus, Milan, Italy).

### Immunofluorescence staining

Twenty-one days after EAE induction, mice were sacrificed, the L4/L5 segments of the spinal cord were exposed from the lumbovertebral column via laminectomy and identified by tracing the dorsal roots from their respective dorsal root ganglia. Formalin-fixed cryostat sections (10 μm) were washed 3 × phosphate-buffered saline (PBS), 0.3% Triton X-100 for 5 min and then were incubated, at room temperature, for 1 h in blocking solution (PBS, 0.3% Triton X-100, 5% albumin bovine serum; PBST). Slices were incubated overnight at 4°C in PBST containing rabbit primary antisera. The primary antibody used were directed against cluster of differentiation 3 (CD3; rabbit, 1:100 dilution) for T-cell co-receptor staining, chemokine (C-C motif) ligand 5 (CCL5; rabbit, 1:100 dilution) for RANTES (Regulated upon Activation Normal T cell Expressed and Secreted) staining, ionized calcium binding adapter molecule 1 (Iba1; rabbit, 1:1000 dilution) for microglial staining, glial fibrillary acidic protein (GFAP; rabbit, 1:1000 dilution) for astrocytes staining. The following day, slides were washed 3 × PBS, 0.3% Triton X-100 for 5 minutes and then sections were incubated in goat anti-rabbit immune globulin G (IgG) secondary antibody labelled with Alexa Fluor 568 (1:500 dilution) and 4',6-diamidino-2-phenylindole (DAPI; 1:2000 dilution), a nuclei marker, in PBST at room temperature for 2 h, in the dark. After 3 × PBS 0.3% Triton X-100 wash for 10 min, slices were mounted using ProLong Gold as a mounting medium. Staining procedures were in accord with a previously published method [28]. Negative control sections (no exposure to the primary antisera) were processed concurrently, in order to exclude the presence of non-specific immunofluorescent staining or cross-immunostaining.

Images were acquired by using an Olympus BX63 microscope equipped with an Olympus XM10 camera and coupled to *CellSense Dimension* Software (Olympus, Milan, Italy). Quantitative analysis of CD3-, Iba1- and GFAP-positive cells was performed by collecting three independent fields through a 20X 0.40NA objective of each mouse spinal cord. Positive cells were counted using the “cell counter” plugin of ImageJ (NIH, Bethesda, MD, USA). Quantitative analysis of CCL5 positive area was performed by collecting three independent fields through a 20X 0.40NA objective of each mouse spinal cord. CCL-5 positive areas were measured using the “threshold” function of ImageJ; data were expressed as the fluorescent area (μm^2^)/mm^2^ of section.

### Release studies

Purified synaptosomes were prepared by homogenizing the cortex, the hippocampi and the spinal cord [29] of control and EAE mice in 10 volumes of 0.32 M sucrose, buffered to pH 7.4 with Tris-(hydroxymethyl)-amino methane [Tris, final concentration (f.c.) 0.01 M] [30]. The homogenate was centrifuged at 1,000 x g for 5 min, and the supernatant was stratified on a discontinuous Percoll gradient (2%, 6%, 10% and 20% v/v in Tris-buffered sucrose) and centrifuged at 33,500 x g for 5 min. The layer between 10% and 20% Percoll (synaptosomal fraction) was collected and washed by centrifugation [31]. The synaptosomal pellets were resuspended in a physiological solution with the following composition (mM): NaCl, 140; KCl, 3; MgSO_4_, 1.2; CaCl_2_, 1.2; NaH_2_PO_4_, 1.2; NaHCO_3_, 5; HEPES, 10; glucose, 10; pH 7.2–7.4.

Synaptosomes were incubated for 15 min a 37°C in a rotary water bath to equilibrate the system and identical portions of the synaptosomal suspensions were layered on microporous filters at the bottom of parallel chambers in a Superfusion System ([32]; Ugo Basile, Comerio, Varese, Italy; [33]) and maintained at 37°C. Synaptosomes were transiently (90 sec) exposed, at *t* = 39 min, to high KCl containing medium (12 mM or 15 mM, as appropriate, NaCl substituting for an equimolar concentration of KCl). Fractions were collected as follow: two 3-min fractions (basal release), one before (*t* = 36–39 min, b1) and one after (*t* = 45–48 min, b2) a 6-min fraction (*t* = 39–45 min; evoked release). Superfusion was always performed with media containing 50 μM amino-oxyacetic acid to avoid metabolism of GABA. Fractions collected were analysed for endogenous glutamate and GABA contents (see below). Synaptosomal protein contents were determined with BCA kit. The amount of endogenous amino acid from synaptosomes in each superfusate fractions was expressed as picomoles per milligram of protein (pmol/mg protein). The K^+^-induced overflow of endogenous glutamate and GABA from synaptosomes was estimated by subtracting the neurotransmitter content into the first and the third fractions collected (basal release, b1 and b3) from that in the 6-min fraction collected during and after the depolarization pulse (evoked release, b2).

#### Endogenous amino acid determination

Collected fractions were analyzed for the endogenous neurotransmitter content. Endogenous glutamate and GABA were measured by high-performance liquid chromatography analysis after precolumn derivatization with *o*-phthalaldehyde and separation on a C18 reverse-phase chromatographic column (10×4.6 mm, 3 μm; at 30°C; Chrompack, Middleburg, The Netherlands) coupled with fluorimetric detection (excitation wavelength, 350 nm; emission wavelength, 450 nm). Buffers and the gradient program were described elsewhere [34]. Homoserine was used as internal standard.

#### Calculations and statistical analysis

Analysis of variance was performed by anova followed by Dunnett’s test or Newman Keuls multiple-comparisons test as appropriate; direct comparisons were performed by Student’s *t*-test. The statistical difference between the untreated and treated EAE mice cumulative score was analyzed by applying the non-parametric Wilcoxon analysis. Data were considered significant for *p* < 0.05 at least.

### Reagents

Pertussis toxin and the Freund’s incomplete adjuvant, Solvent Blue 38, Periodic Acid were acquired from Sigma-Aldrich (Saint Louis, MO, USA). Pierce^™^ BCA Protein Assay Kit was from Thermo Fisher Scientific 168 Third Avenue Waltham, MA USA 02451. Myelin oligodendrocyte glycoprotein (MOG) was purchased from Espikem (Florence, Italy). *Mycobacterium tuberculosis* (H37Ra) was obtained from DIFCO BACTO Microbiology (Lawrence, KA, USA). Fingolimod was supplied by Novartis Pharma AG, Basel, Switzerland. The anti-rabbit Alexa Fluor 568 was acquired from Molecular Probes, Invitrogen (Carlsbad, CA, USA). Schiff ‘s reagent is from Bio-optica (Milan, Italy). Primary antibody against CD3 was from SP Clone (Pleasanton, CA, USA). Primary antibody against CCL5 was from Abcam (Cambridge, UK) ionized calcium binding adapter molecule 1 (Iba1; rabbit, was from Wako (Richmond, VA, USA). Primary antibody against Anti Iba1; rabbit, was from Wako (Richmond, VA, USA). Primary antibody against glial fibrillary acidic protein (GFAP; rabbit) was from DAKO, (Carpinteria, CA, USA). Primary antibody against cluster of differentiation 3 (CD3; rabbit) was from SP Clone (Pleasanton, CA, USA). DAPI (4',6-diamidin-2-fenilindolo) and ProLong Gold were from Life Technologies-Thermo scientific, (Rockford, IL, USA).

## Results

### Prophylactic fingolimod reduces the severity of clinical signs and anxiety-related behaviour in EAE mice

**Fig 1:**
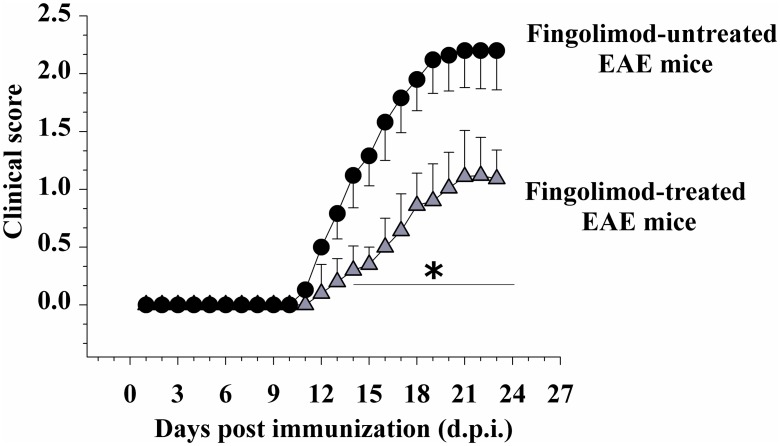
Effect of *in vivo* prophylactic fingolimod on the clinical score of EAE mice. Animal scores in untreated (black circle, n = 22 animals) and fingolimod (0.3 mg/kg) treated (grey triangle, n = 22 animals) EAE mice at different stages of disease. Clinical signs were detected daily in EAE mice and are expressed as average (mean ± SEM * *p* < 0.05 at least versus daily clinical score in untreated EAE mice.

Control [non-immunized, MOG_35-55_ (-)] and EAE [immunized, MOG_35-55_ (+)] female C57JBl mice were randomly assigned to the following groups: fingolimod-untreated control, fingolimod (0.3 mg/kg)-treated control, fingolimod-untreated EAE, or fingolimod (0.3 mg/kg)-treated EAE mice. Clinical signs in fingolimod-untreated EAE mice became evident at 12 ± 1 d.p.i. and developed a rapid worsening that peaked around 20 ± 1 d.p.i. (clinical score = 2.12 ± 0.29, *n* = 22 animals), when a chronic phase was reached (Fig 1). Similarly, in prophylactically fingolimod-treated EAE mice, clinical symptoms were observed starting from 12 ± 1 d.p.i. as well, but they were less pronounced, with the maximal gravity (clinical score = 1.11 ± 0.40, *n* = 22 animals, p < 0.05 at least versus untreated EAE mice, Fig 1) being evident starting from 21 d.p.i. ± 1. The total clinical score in untreated EAE mice amounted to 20.03 ± 0.92 (*n* = 22) and in fingolimod-treated mice to 8.18 ± 0.78 (*n* = 22, *p* < 0.05 at least versus untreated EAE mice, -59.16%). These observations are consistent with previously published findings, which showed a drastic reduction of the clinical gravity in EAE mice orally administered fingolimod (fingolimod (0.3 mg/kg) administered by oral gavage [21]; fingolimod (0.5 mg/kg) dissolved in the drinking water [24]).

### Prophylactic fingolimod reduces demyelination and inflammation in EAE mice spinal cord

Prophylactically administered oral fingolimod was also shown to reduce inflammation and demyelination in the spinal cord of EAE mice [35]. In order to further support the appropriateness of the protocol applied in our study, experiments were dedicated to analyse the impact of prophylactic fingolimod (0.3 mg /kg) on inflammation and demyelination on the above-mentioned histopathological hallmarks.

**Fig 2:**
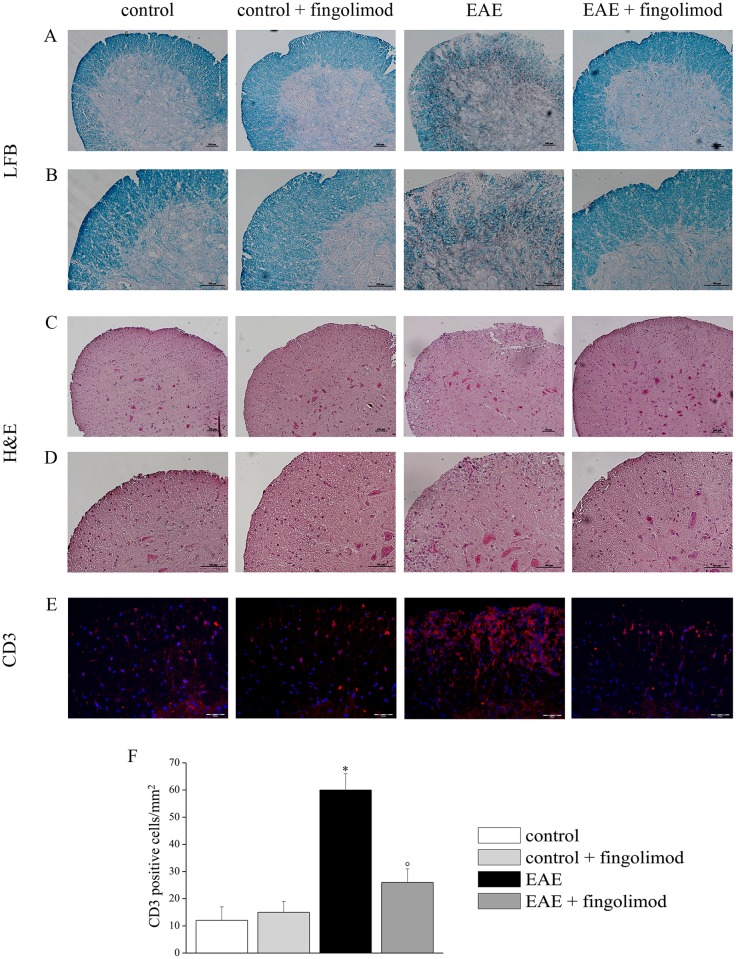
Effects of *in vivo* prophylactic fingolimod on demyelination and inflammation in the spinal cord of EAE mice at the acute stage of disease. On day 21 post EAE induction, spinal cord sections were analyzed. (**A, B**) Luxol Fast Blue stained myelin in blue and revealed demyelinated areas; (**C, D**) Hematoxylin/Eosin stained infiltrated cells and tissue components. Representative images at 10X (**A**, **C**) and 20X (**B**, **D**) magnification of white matter of anterior spinal cord of each group are shown. (**E**) CD3-positive cells in spinal cord. Sections were immune-stained with the anti-CD3 antibody (red) to recognize T-cells and with DAPI (blue) to identify cell nuclei. Representative images of 20X magnification of the spinal cord section. (**F**) The number of CD3-positive cells/mm^2^ in the spinal cord of mice of each treatment-group is reported. * *p* < 0.05 *versus* control untreated mice; ° *p* < 0.05 *versus* untreated EAE mice. Histochemical analysis and CD3 staining were performed on slices from control untreated mice (n = 5), from control 0.3 mg /kg fingolimod-treated mice (n = 4), from EAE untreated mice (n = 5), from EAE 0.3 mg /kg fingolimod-treated mice (n = 5).

Luxol Fast Blue and Hematoxylin/Eosin stainings of the spinal cord of control, fingolimod (0.3 mg/kg)-treated control, EAE, and fingolimod (0.3 mg/kg)-treated EAE mice were performed to evaluate myelin distribution and inflammation, respectively. Twenty-one days after EAE induction, demyelination was clearly evident in the white matter of spinal cord (Fig 2, panels A and B), but not in EAE animals treated with fingolimod, where myelin showed a density comparable to that of controls. Moreover, the cell infiltration was largely prevented in the spinal cord white matter in fingolimod-treated EAE mice (Fig 2, panels C and D) when compared to the control groups(s).

In order to evaluate the characteristics of the infiltrated cells, T-cells were stained with anti-CD3 antibody [Fig 2E, in red, DAPI (blue) to identify cell nuclei]. In EAE mice, CD3-positive cells were strongly present in comparison to fingolimod-untreated and treated control animals, while in fingolimod-treated EAE the number decreased up to the control value (Fig 2F).

### Prophylactic fingolimod reduces anxiety-related behaviour in EAE mice

**Fig 3:**
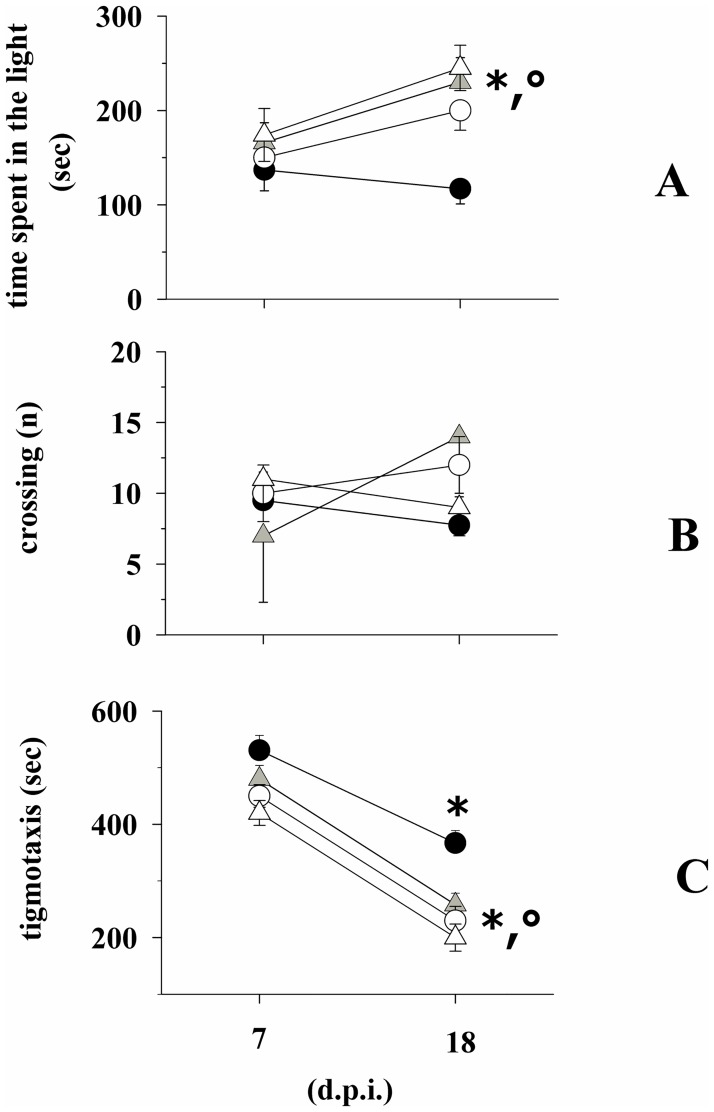
Effect of *in vivo* prophylactic fingolimod on the anxiety-like behaviour in EAE mice. Fingolimod untreated (white circle, n = 15 mice) control mice; fingolimod treated (white triangle, n = 15 mice) control mice; Fingolimod untreated (black circle, n = 16 mice) EAE mice; Fingolimod treated (grey triangle, n = 16 mice) EAE mice at 7 and 18 d.p.i. were monitored for anxiety-therapeutic behaviour that was quantified in the light dark box as total time spent into the lighted compartment [time in the light (sec), **A**] and as number of crossings from the lighted to the dark side of the box [crossings (n), **B**]. Anxiety was also quantified as time spent in periphery (thigmotaxis) in the open field (**C**). Data represent the media ± SEM. * *p* < 0.05 at least versus fingolimod-treated EAE mice at 7 d.p.i; ° *p* < 0.05 at least versus untreated EAE mice at 18 d.p.i.

Behavioural tests were carried out in EAE mice at 7 d.p.i. (*i*.*e*., before the start of drug treatment) and at 18 d.p.i., (3 days before the mice sacrifice, when an almost maximal gravity of clinical symptoms is observed in EAE mice) to quantify the effects of fingolimod on anxiety-like behaviour in EAE mice. At 7 d.p.i., fingolimod-untreated and -treated EAE mice spent comparable amounts of time in the lighted side of the box (Fig 3A). Differently, at 18 d.p.i., the time spent in the lighted side of the box by the fingolimod-treated EAE mice was significantly higher than that spent by the untreated EAE mice. Inasmuch, this value was significantly higher than that recorded at 7 d.p.i., suggesting that the prolonged administration of fingolimod had reduced the anxiety during disease progression. The time spent by untreated EAE mice in the lighted side of the box at 18 d.p.i. was not significantly modified when compared to the value observed at 7 d.p.i. (Fig 3A). Significant changes in the number of crossings from the lighted to the dark side of the box for both the untreated and the fingolimod-treated EAE mice were not observed either at 7 d.p.i. or at 18 d.p.i (Fig 3B). Finally, thigmotaxis for EAE mice at 18 d.p.i. was significantly reduced when compared to untreated EAE mice (Fig 3C). Of note, for both fingolimod-untreated and treated EAE mice, the thigmotaxis values at 18 d.p.i were significantly reduced when compared to those observed at 7 d.p.i., suggesting that adaptation might have occurred in mice exposed twice to the behavioural test.

### Prophylactic fingolimod ameliorates glutamate and GABA exocytosis in selected regions of central nervous system of EAE mice at the acute stage of disease

Synaptosomes isolated from the cortex, the hippocampus, and the spinal cord of control, fingolimod-treated control, EAE (21 ± 1 d.p.i.), and fingolimod-treated EAE mice (21 ± 1 d.p.i.) were superfused and the spontaneous and the depolarization-evoked release of endogenous glutamate and GABA was monitored. In basal conditions, *i*.*e*. before the depolarizing stimulus was applied, the release of endogenous glutamate was unmodified in synaptosomes isolated from the cortex, the hippocampus and the spinal cord (Table 1) of animals belonging to the four groups. Table 1 shows that a comparable amount of endogenous glutamate and GABA in the b1 (basal release) fractions was detected.

**Table 1:** Prophylactic fingolimod does not modify the spontaneous release of endogenous glutamate and GABA from synaptosomes isolated from selected regions of central nervous system of EAE mice at the acute stage of disease.

	Control mice	Fingolimod-treated control mice	EAE mice	Fingolimod treated EAE mice
	endogenous glutamate (pmoles / mg protein)			
cortex	123.4 ± 13.7	132.7 ± 16.0	108.4 ± 15.3	128.1 ± 15.2
hippocampus	155.9 ± 17.3	172.6 ± 22.4	182.1 ± 21.33	162.8 ± 16.3
spinal cord	198.3 ± 22.5	212.4 ± 21.3	209.7 ± 18.4	203.5 ± 22.1
	endogenous GABA (pmoles / mg protein)			
cortex	83.2 ± 11.2	78.9 ± 14,5	99.5 ± 13.5	89.4 ± 16.7
hippocampus	75.4 ± 12.9	62.3 ± 9.5	71.5 ± 11.3	78.2 ± 8.3
spinal cord	65.7 ± 13.5	72.4 ± 9.1	58.4 ± 11.4	78.3 ± 14.3

Synaptosomes isolated from the cortex, the hippocampus and the spinal cord of control and EAE mice administered with fingolimod (0.3 mg / kg, for 14 days, prophylactically administered in the drinking water) were stratified at the bottom of superfusion chambers and superfused as described in the Method Section. The amount of endogenous glutamate and GABA in the first 3-min fraction collected before the depolarizing stimulus was applied (b1, basal release) was quantified. Results are expressed as pmoles / mg protein. Data are the media ± SEM of values from at least four different experiments run in triplicate.

**Fig 4:**
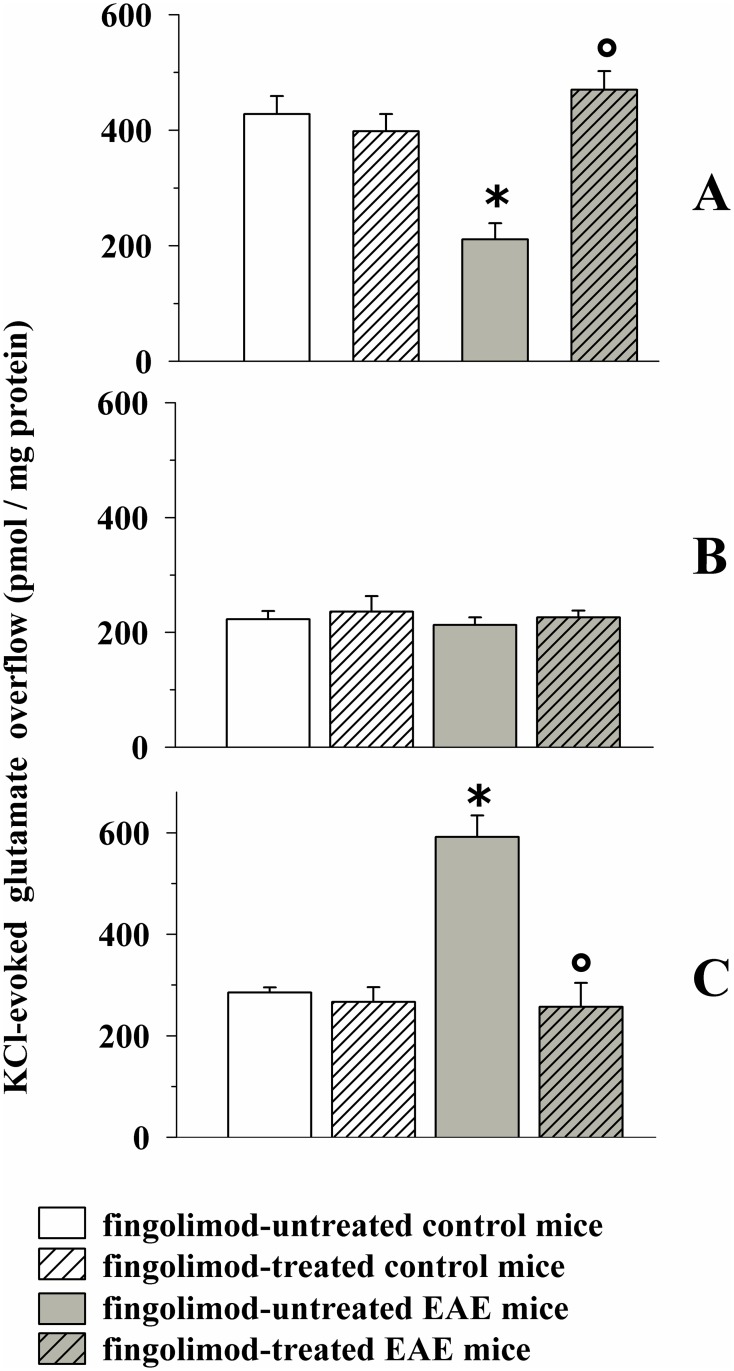
Effect of *in vivo* prophylactic fingolimod (0.3 mg/kg) on the depolarization-evoked exocytosis of endogenous glutamate from nerve terminals isolated from selected regions of the CNS of control and EAE mice. Female mice were randomly assigned to the following groups: control mice (empty bar, n = 8 mice); fingolimod-treated control mice (rising-right hatched empty bar, n = 8 mice); EAE mice (grey bar, n = 8 mice); fingolimod-treated EAE mice (rising-right hatched grey bar, n = 8 mice). Fingolimod was administered in the drinking water for 14 days starting from 7 d.p.i. At 21 ± 1 d.p.i mice were sacrificed and cortical (**A**), hippocampal (**B**) and spinal cord (**C**) synaptosomes were isolated to monitor the exocytosis of endogenous glutamate elicited by a mild (12 mM KCl enriched superfusion medium for cortical and hippocampal synaptosomes and 15 mM KCl enriched superfusion medium for spinal cord synaptosomes) depolarizing stimulus. Results are expressed as KCl-evoked overflow; data are expressed as pmoles / mg protein and represent the mean ± SEM. Each experiment was carried out with the synaptosomal preparations isolated from one animal for each group, and it was run in triplicate (three superfusion chambers for each animal). * *p* < 0.05 at least versus control untreated mice; ° *p* < 0.05 at least versus EAE untreated mice.

Fig 4A shows that the chronic administration of fingolimod (0.3 mg/kg, 14 days treatment) did not significantly modify the 12 mM KCl-evoked release of endogenous glutamate from control mice. Differently, the 12 mM KCl-evoked release of endogenous glutamate from the cortex of EAE mice was significantly reduced when compared to control mice [8,9]. Finally, the 12 mM KCl-evoked release of endogenous glutamate from cortical synaptosomes isolated from fingolimod-treated EAE mice was significantly higher than that detected from EAE mice, but did not significantly differ from to that observed from control fingolimod-treated mice.

As to the hippocampus, significant changes in the 12 mM KCl-evoked endogenous glutamate outflow from nerve terminals isolated from EAE mice were not observed when compared to control mice. Inasmuch, prophylactic fingolimod failed to significantly modify glutamate exocytosis from both control and EAE hippocampal nerve endings (Fig 4B).

Finally, the 15 mM KCl-evoked overflow of endogenous glutamate from the nerve terminals isolated from the spinal cord of EAE mice at 21 d.p.i. was significantly increased when compared to that observed from control mice [8]. Prophylactic fingolimod, inactive in control mice, drastically reduced glutamate exocytosis in spinal cord synaptosomes from EAE mice. The 15 mM KCl-evoked glutamate outflow from fingolimod-treated EAE mice did not differ glutamate exocytosis from control mice (Fig 4C).

**Fig 5:**
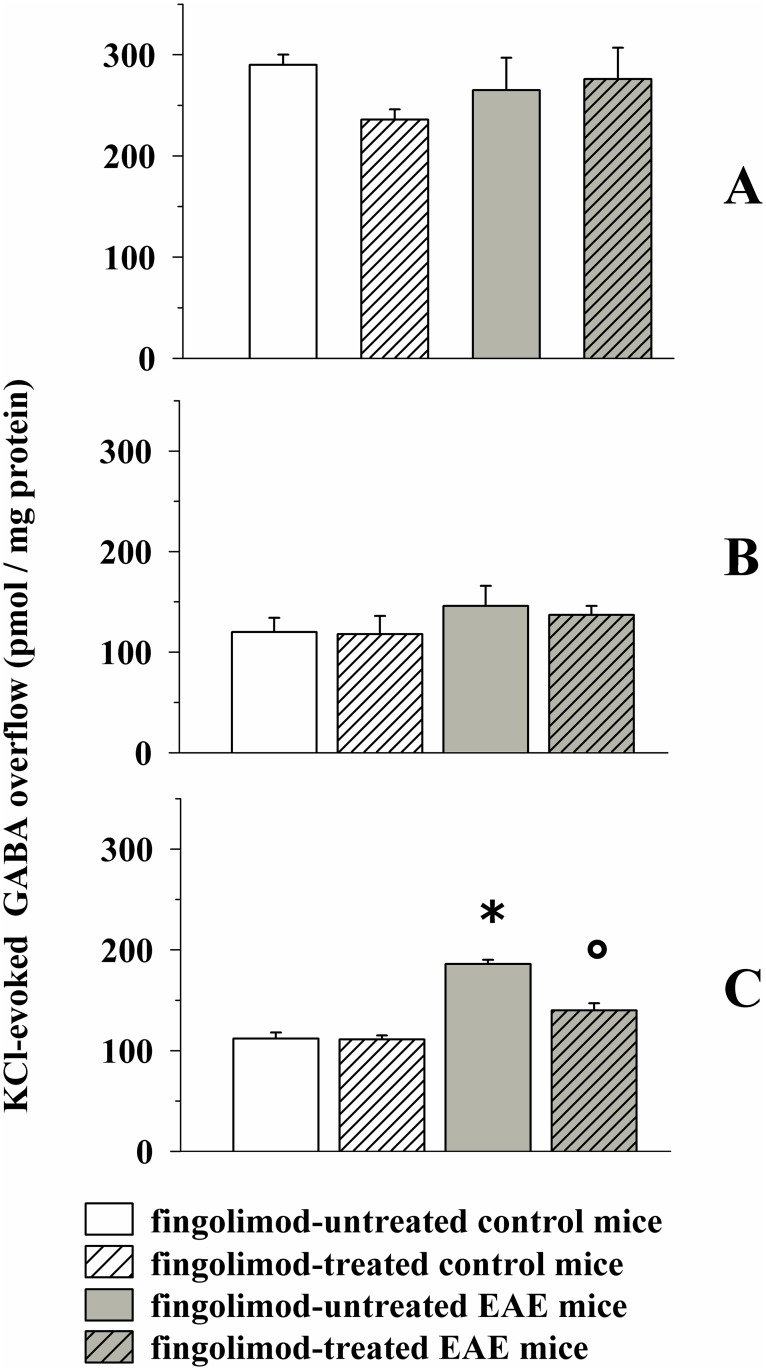
Effect of *in vivo* prophylactic fingolimod (0.3 mg/kg) on the depolarization-evoked exocytosis of endogenous GABA from nerve terminals isolated from selected regions of the CNS of control and EAE mice. Female mice were randomly assigned to the following groups: control mice (empty bar, n = 8 mice); fingolimod-treated control mice (rising-right hatched empty bar, n = 8 mice); EAE mice (grey bar, n = 8 mice); fingolimod-treated EAE mice (rising-right hatched grey bar, n = 8 mice). Fingolimod was administered as described above. At 21 ± 1 d.p.i. mice were sacrificed and cortical (**A**), hippocampal (**B**) and spinal cord (**C**) synaptosomes were isolated to monitor the exocytosis of endogenous GABA elicited by a mild depolarizing stimulus as previously described. Results are expressed as KCl-evoked overflow; data are expressed as pmoles / mg protein and represent the mean ± SEM. * *p* < 0.05 at least versus control untreated mice; ° *p* < 0.05 at least versus EAE untreated mice.

The depolarization-evoked release of endogenous GABA was unmodified in both cortical and hippocampal synaptosomes of EAE mice when compared to control (Fig 5A and 5B), but it was significantly augmented in spinal cord nerve terminals isolated from EAE mice at 21 ± 1 d.p.i. (Fig 5C).

Prophylactic fingolimod failed to modify GABA exocytosis from cortical and hippocampal synaptosomes isolated from both control and EAE mice, but significantly reduced the 15 mM KCl-evoked exocytosis of GABA from the spinal cord nerve endings of EAE mice (Fig 5C).

### Effects of prophylactic lower doses of fingolimod on glutamate and GABA exocytosis in selected regions of central nervous system of EAE mice at the acute stage of disease

**Fig 6:**
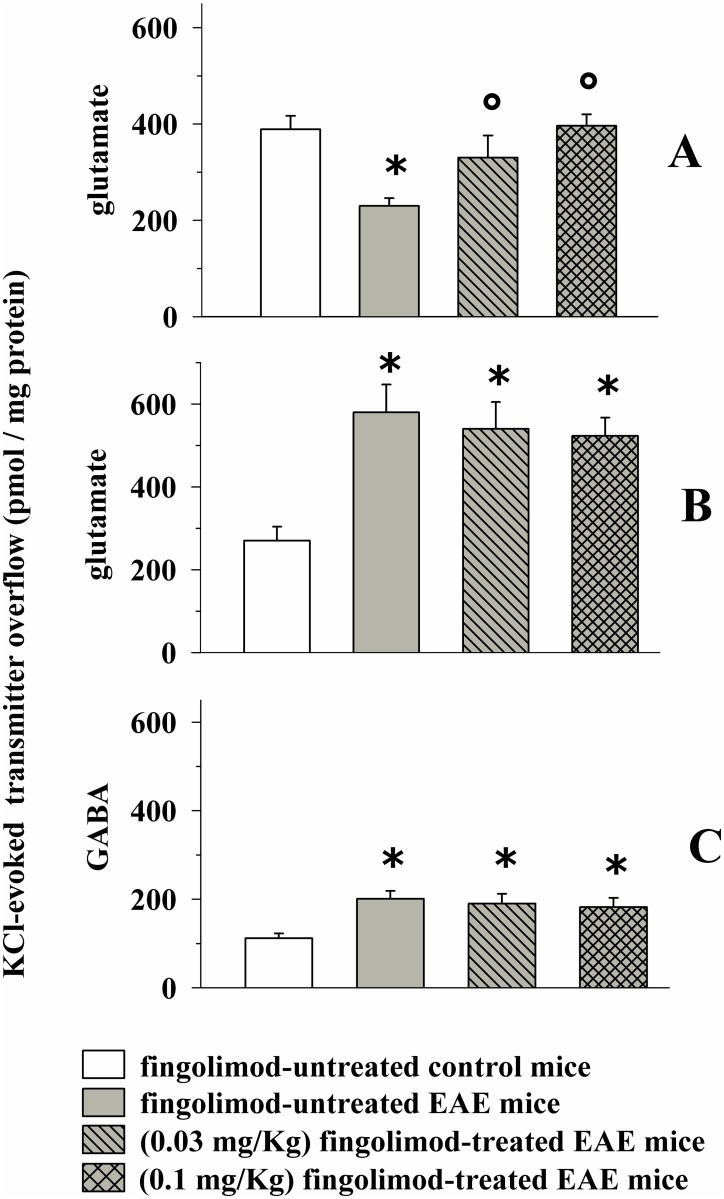
Effect of *in vivo* different doses of prophylactic fingolimod on the depolarization-evoked exocytosis of endogenous glutamate and GABA from nerve terminals isolated from selected regions of the CNS of control and EAE mice. Female mice were randomly assigned to the following groups: control mice (empty bar, n = 6 mice); EAE mice (grey bar, n = 8 mice); fingolimod (0.03 mg/kg, n = 8 mice)-treated EAE mice (rising-left hatched grey bar); fingolimod (0.1 mg/kg)-treated EAE mice (cross-hatched grey bar). Fingolimod was administered as described above. At 21 ± 1 d.p.i. mice were sacrificed and cortical (A) and spinal cord (**B** and **C**) synaptosomes were isolated to monitor the exocytosis of endogenous glutamate (**A** and **B**) and GABA (**C**) elicited by a mild depolarizing stimulus as previously described. Results are expressed as KCl-evoked overflow of the endogenous aminoacids; data are expressed as pmoles / mg protein and represent the mean ± SEM experiments run in triplicate. * *p* < 0.05 at least versus control untreated mice; ° *p* < 0.05 at least versus EAE untreated mice.

As illustrated in Fig 6A *in vivo* chronic (14 days) 0.1 and 0.03 mg/kg fingolimod significantly recovered, although to a different extent, glutamate exocytosis from cortical synaptosomes of EAE mice at 21 ± 1 d.p.i. Conversely, neither 0.03 nor 0.1 mg/kg fingolimod reduced glutamate exocytosis from spinal cord synaptosomes of EAE mice at the acute stage of disease (Fig 6B). Since at this stage of disease the high K^+^-evoked glutamate exocytosis from EAE mouse hippocampal synaptosomes was unaltered when compared to control mice, the impact of *in vivo* low doses of fingolimod in this CNS region was not investigated. Finally, neither 0.1 nor 0.03 mg/kg fingolimod restored GABA exocytosis to physiological level in spinal cord EAE mouse nerve terminals (Fig 6C). The impact of fingolimod on the GABA exocytosis from cortical and hippocampal synaptosomes was not investigated since these functional parameters were unaltered in the cortex of EAE mice at 21± 1 d.p.i.. At the administered doses, fingolimod failed to affect the K^+^-evoked glutamate and GABA exocytosis from nerve terminals (not shown).

### Prophylactic fingolimod reduces glial cell activation in EAE mice spinal cord

In recent years, the role of astrocytes and microglial cells in controlling glutamate transmission in CNS has emerged. In particular, by either recapturing excessive glutamate or by releasing it both cell types could drive the chemical force(s) accounting for neuropathological features in EAE mice [4]. Experiments dedicated to quantify astrocytosis and activation of microglia were carried out in the CNS region where an overt pathological increase in glutamate exocytosis was observed, *i*.*e*., in the spinal cord of EAE mice at the acute (i.e. 21 d.p.i.) stage of disease, in an attempt to clarify whether and to what extent prophylactic fingolimod (0.3 mg / kg) affected these parameters.

**Fig 7:**
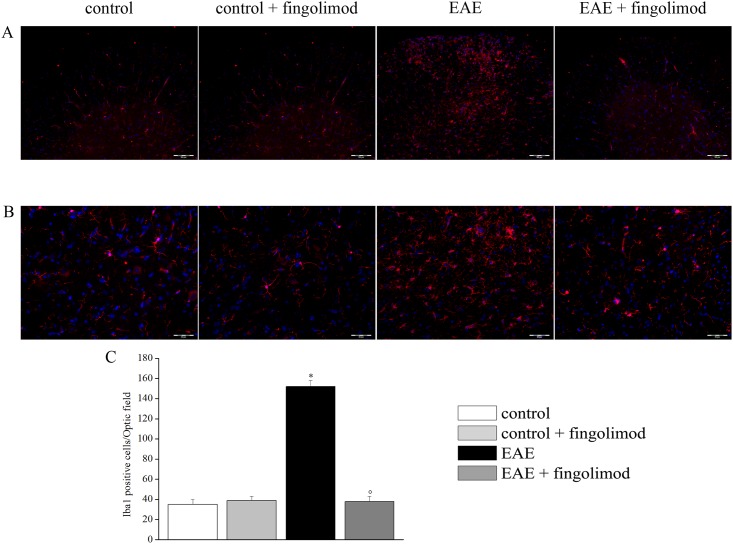
Effects of *in vivo* prophylactic fingolimod on microglial cells in the spinal cord of EAE mice at the acute stage of disease. On day 21 post EAE induction, tissue sections were immuno-stained with the anti-Iba1 antibody (red) to recognize microglia cells and with DAPI (blue) to identify cell nuclei. (**A**) 10X: Low-magnification image of spinal cord sections. (**B**) 20X: High-magnification image of the spinal cord sections. (**C**) Quantitative evaluation of the number of Iba1-positive cells/Optic field in the spinal cord of mice of each treatment-group. * *p* < 0.05 *versus* all other groups; ° *p* < 0.05 versus untreated EAE mice.

**Fig 8:**
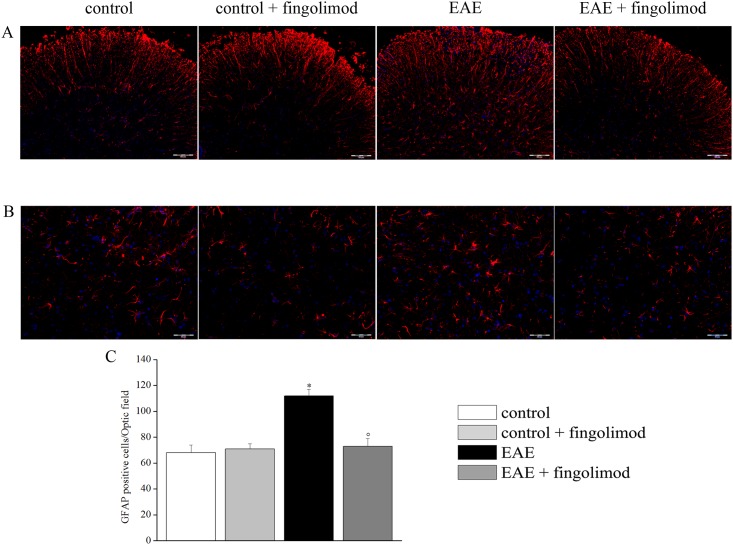
Effects of *in vivo* prophylactic fingolimod on astrocytes in the spinal cord of EAE mice at the acute stage of disease. On day 21 post EAE induction, tissue sections were immuno-stained with the anti-GFAP antibody (red) to recognize astrocytes and with DAPI (blue) to identify cell nuclei. (**A**) 10X: Low-magnification image of spinal cord sections. (**B**) 20X: High-magnification image of the spinal cord sections. (**C**) Quantitative evaluation of the number of GFAP-positive cells/Optic field in the spinal cord of mice of each treatment-group. * *p* < 0.05 *versus* all other groups; ° *p* < 0.05 versus untreated EAE mice.

Spinal cord slices were immunolabelled with Iba-1 or GFAP antibodies to analyze, respectively, microglia (Fig 7) and astrocytes (Fig 8). Iba1 positive cells exhibited small bodies and finely branched processes in both untreated and fingolimod-treated control animals. In EAE mice, the morphology of Iba1 positive cells was modified in a mild activation phenotype with characteristic thickened processes. In fingolimod-treated EAE mice, cell morphology appeared similar to that of control animals (Fig 7, panel A and B). The number of Iba1 positive cells in EAE group was increased in comparison to control, whereas in fingolimod-treated EAE mice microglia density was comparable to that observed in control (Fig 7, panel C).

As depicted in Fig 8, GFAP-positive cells showed typical non-reactive astrocyte morphology in both untreated and fingolimod treated control animals. On the contrary, after EAE induction, astrocytes were reactive and hypertrophic. In fingolimod-treated EAE mice, astrocyte morphology appeared similar to that of control animals (Fig 8, panels A and B). Numerically, GFAP-positive cells were increased in EAE mice in comparison to other groups. Fingolimod prevented the EAE-dependent astrocyte density enhancement, whereas the compound did not affect the cell number in control animals (Fig 8, panel C).

### Prophylactic fingolimod reduces the endogenous overexpression of CCL5 in EAE mice spinal cord

**Fig 9:**
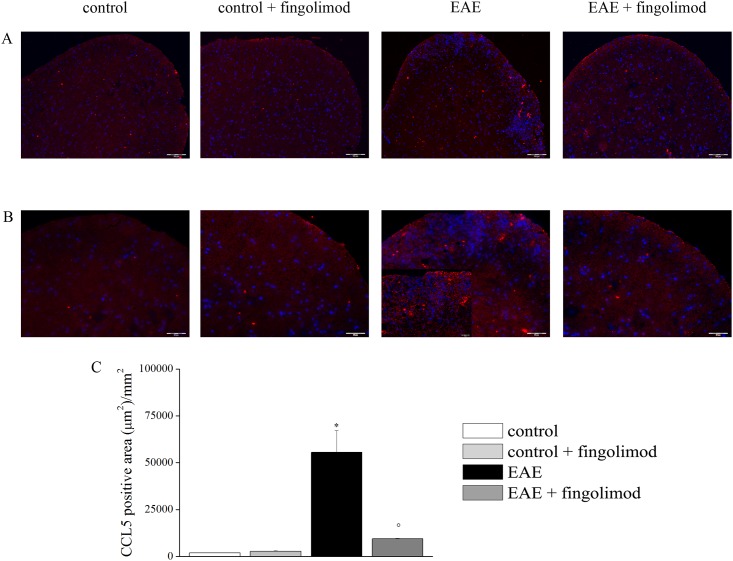
Effects of *in vivo* prophylactic fingolimod on CCL5 in the spinal cord of EAE mice at the acute stage of disease. On day 21 post EAE induction, tissue sections were immuno-stained with the anti-CCL5 antibody (red) and with DAPI (blue) to identify cell nuclei. (**A**) 10X: Low-magnification image of spinal cord sections; (**B**) 20X: High-magnification image of the spinal cord sections; (**C**) The CCL5 positive area (μm^2^)/mm^2^ in the spinal cord of mice of each treatment-group is reported. * *p* < 0.05 *versus* control untreated mice; ° *p* < 0.05 *versus* untreated EAE mice.

Previous studies have shown an abnormal overexpression of the chemokine CCL5 in the spinal cord of EAE mice, that was proposed to have a role in determining altered glutamate exocytosis at nerve terminals in this CNS region [9,10]. The expression of the inflammatory chemokine CCL5 was measured in the spinal cord of both fingolimod untreated and treated EAE mice and respective control by immunofluorescence staining (Fig 9, panels A and B). Fig 9 shows that twenty-one days after EAE induction, the CCL5-stained area in spinal cord slices from EAE mice significantly increased in comparison to the control animals. Oral prophylactic fingolimod prevented the CCL5 enhancement in spinal cord slices, leaving the endogenous production of CCL5 in spinal cord slices from control mice unchanged (Fig 9C).

### Therapeutic fingolimod reduces the severity of clinical signs in EAE mice

**Fig 10:**
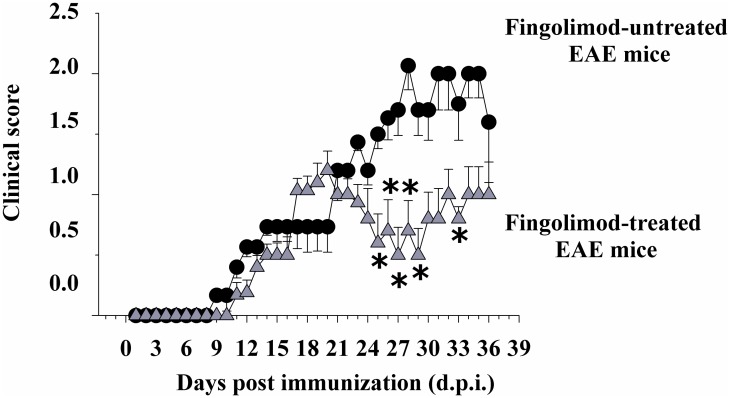
Effect of *in vivo* therapeutic fingolimod on the clinical score of EAE mice. Animal scores in control (untreated, black circle, n = 16 animals) and fingolimod (0.3 mg/kg, starting from 21 d.p.i. until 35 d.p.i.)-treated (grey triangle, n = 15 animals) EAE mice at different stages of disease. Clinical signs were detected daily in EAE mice and are expressed as average (mean ± SEM). * *p* < 0.05 at least versus daily clinical score in untreated EAE mice.

The course of disease symptoms was monitored in EAE mice chronically administered fingolimod (0.3 mg/kg) starting from 21 d.p.i. till 35 d.p.i. Fig 10 shows that the clinical score in fingolimod-administered EAE mice was significantly modified when compared to untreated EAE mice starting from 25 ± 1 d.p.i. and did not worsen until 35 ± 1 d.p.i. The total clinical score in untreated EAE mice amounted to 30.68 ± 2.42 (*n* = 16) and in fingolimod-treated mice to 12.18 ± 1.58 (*n* = 15, *p* < 0.05 at least versus untreated EAE mice, -60.30%).

### Therapeutic fingolimod ameliorates glutamate and GABA exocytosis in selected regions of central nervous system of EAE mice after the acute stage of disease

**Fig 11:**
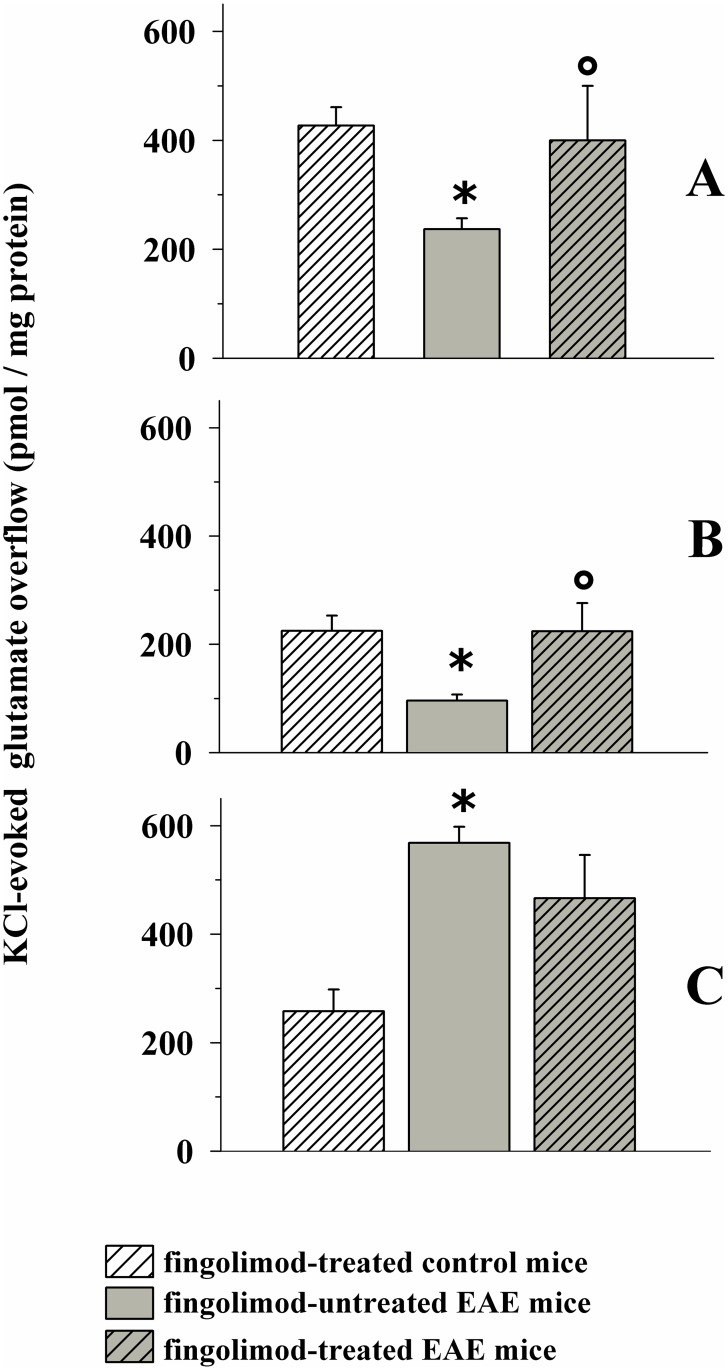
Effect of *in vivo* therapeutic fingolimod (0.3 mg/kg) on the depolarization-evoked exocytosis of endogenous glutamate from nerve terminals isolated from selected regions of the CNS of control and EAE mice. Female mice were randomly assigned to the following groups: fingolimod-treated control mice (rising-right hatched empty bar, n = 6 animals); EAE mice (grey bar, n = 10 animals); fingolimod-treated EAE mice (rising-right hatched grey bar, n = 10 animals). Fingolimod was administered in the drinking water for 14 days starting from 21 d.p.i.. At 35 ± 1 d.p.i. mice were sacrifice and cortical (A), hippocampal (**B**) and spinal cord (**C**) synaptosomes were isolated to monitor the exocytosis of endogenous glutamate elicited by a mild depolarizing stimulus as previously described. Results are expressed as KCl-evoked overflow; data are expressed as pmoles / mg protein and represent the mean ± SEM. * *p* < 0.05 at least versus fingolimod-treated control mice; ° *p* < 0.05 at least versus EAE mice.

Fig 11A shows that glutamate exocytosis from cortical nerve terminals from EAE mice at 35 ± 1 d.p.i. was significantly lower than that observed from fingolimod-treated control mice. The figure also shows that *in vivo* chronic therapeutic administration of 0.3 mg/kg fingolimod (from 21 d.p.i. to 35 d.p.i) restored glutamate exocytosis to the level observed in fingolimod-treated control mice.

We then focused on the hippocampal region. Previous observations had shown that glutamate exocytosis from hippocampal nerve endings of EAE mice at the acute stage of disease (i.e. 21 ± 1 d.p.i) was unmodified when compared to control. Quite surprisingly, we found that glutamate exocytosis from nerve terminals isolated from the hippocampus of EAE mice at 35 ± 1 d.p.i was significantly reduced when compared to controls (Fig 11B). Therapeutic fingolimod (0.3 mg/kg) significantly recovered glutamate exocytosis (Fig 11B) to the level observed in fingolimod-treated control mice.

Finally, we investigated the impact of therapeutic fingolimod (0.3 mg/kg) on glutamate exocytosis from spinal cord synaptosomes of EAE mice. Glutamate exocytosis was significantly augmented in EAE mice at 35 ± 1 d.p.i. when compared to control mice. Therapeutic fingolimod (0.3 mg/kg) reduced, although not significantly, the abnormal glutamate exocytosis from EAE mouse spinal cord synaptosomes (Fig 11C). Therapeutic fingolimod did not modify the K^+^-evoked release of glutamate from control mice (not shown).

Experiments were dedicated to study the impact of a lower dose of fingolimod on glutamate release impairments. The oral administration of 0.1 mg /kg fingolimod starting from 21 d.p.i. for 14 days caused a slight, although not significant, amelioration of glutamate release efficiency in the cortex (fingolimod treated control mice: 498 ± 42; EAE mice: 235 ± 28; fingolimod-treated EAE mice: 311 ± 43, results reported as KCl-evoked overflow; data represent the mean ± SEM and are expressed as pmoles / mg protein, n = 4 animals for each groups, *n*.*s*.) as and the hippocampus (fingolimod treated control mice: 249 ± 36; EAE mice: 120 ± 22; fingolimod-treated EAE mice: 189 ± 30, results reported as above, n = 4 animals for each groups, *n*.*s*.).

**Fig 12:**
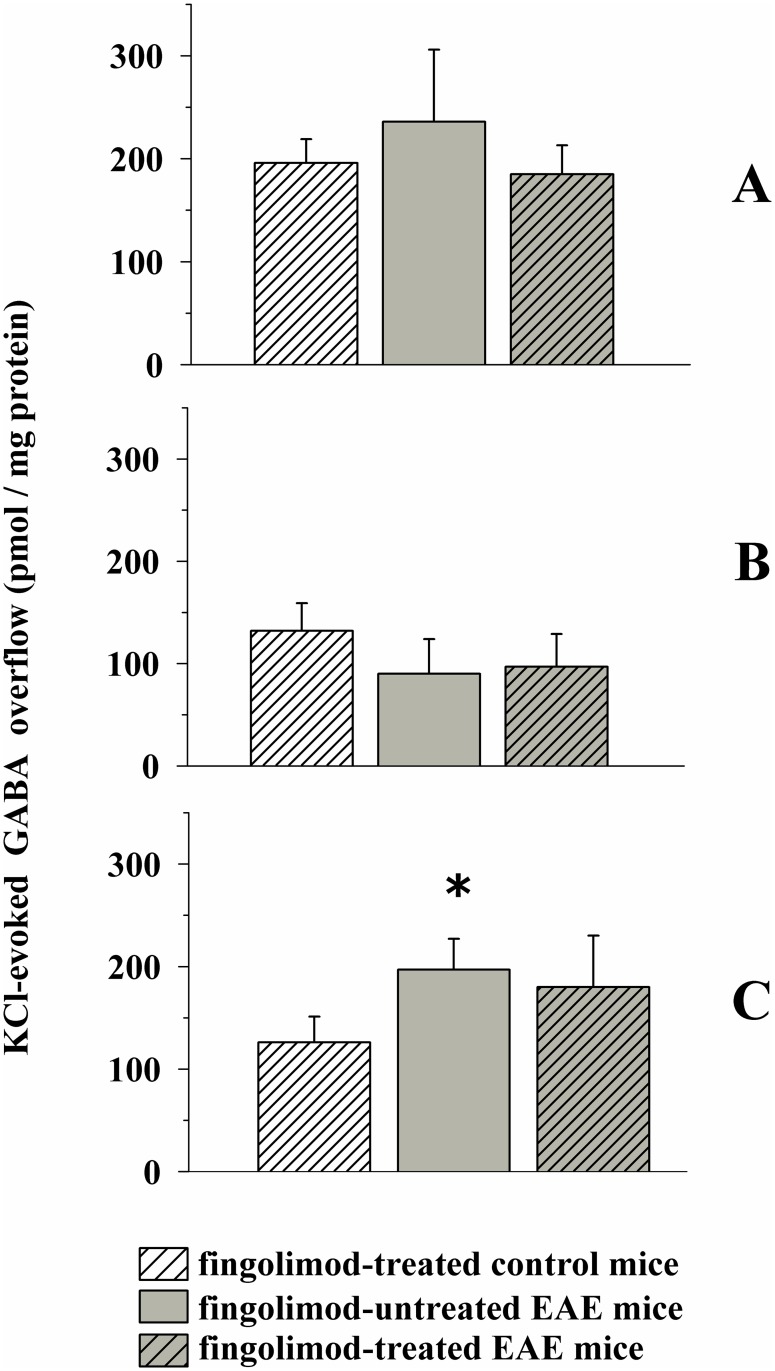
Effect of *in vivo* therapeutic fingolimod (0.3 mg/kg) on the depolarization-evoked exocytosis of endogenous GABA from nerve terminals isolated from selected regions of the CNS of control and EAE mice. Female mice were randomly assigned to the following groups: fingolimod-treated control mice (rising-right hatched empty bar, n = 6 animals); EAE mice (grey bar, n = 10 animals); fingolimod-treated EAE mice (rising-right hatched grey bar, n = 10 animals). Fingolimod was administered in the drinking water for 14 days starting from 21 d.p.i.. At 35 ± 1 d.p.i. mice were sacrifice and cortical (**A**), hippocampal (**B**) and spinal cord (**C**) synaptosomes were isolated to monitor the exocytosis of endogenous GABA elicited by a mild depolarizing stimulus as described above. Results are expressed as KCl-evoked overflow; data are expressed as pmoles / mg protein and represent the mean ± SEM. * *p* < 0.05 at least versus fingolimod-treated control mice.

Significant changes in GABA overflow could not be observed in cortical and hippocampal nerve endings from EAE mice when compared to controls (Fig 12A and 12B). On the contrary, GABA exocytosis was significantly increased from nerve endings isolated from the spinal cord of EAE mice at 35 ± 1 d.p.i. when compared to controls (Fig 12C). Therapeutic 0.3 mg/kg fingolimod failed to recover GABA exocytosis in EAE mice at 35 ± 1 d.p.i (Fig 12C). Therapeutic fingolimod did not modify the K^+^-evoked release of GABA from control mice (not shown).

## Discussion

Our study aimed to investigate the effect of *in vivo* oral fingolimod dissolved in the drinking water on the onset and during the course of EAE in mice. In particular, the study aimed at quantifying the effects of this drug on the presynaptic defects that occurs in selected CNS regions of EAE mice at different stages of disease.

Previous data in the literature had shown that oral fingolimod in the drinking water (0.1 mg/kg, 28 days treatment starting from 15 d.p.i) reduces the incidence and the severity of clinical score in EAE mice, also ameliorating demyelination in the spinal cord [36]. The positive effects of oral fingolimod were also highlighted in a subsequent work showing that fingolimod administered in the drinking water (0.5 mg/kg, 28 days treatment starting from 3 d.p.i; [24]) reduced the gravity of clinical symptoms.

In line with these observations, our results confirm that the prophylactic (0.3 mg/kg, 14 days treatment starting from 7 d.p.i.) administration of fingolimod dissolved in the drinking water reduces the gravity of clinical symptoms in EAE mice. In addition, fingolimod significantly alleviated inflammatory infiltration as well as demyelination in the spinal cord. Finally, a significant amelioration of the anxiety-related behaviour was detected. In this regard, controversial results are present in the literature. Sheridan and coll. [25] demonstrated that the prophylactic oral administration of fingolimod improves motor activity. They also reported positive effects on social and emotional behaviours. Differently, de Bruin and colleagues [24] did not observe improved motor performance in EAE mice administered fingolimod in the drinking water starting from the early stage of disease, while social novelty preference was normalized by late fingolimod treatment. Of note, in the present study significant changes to the spontaneous motor behaviour measured as number of crossing from the dark to the lighted side of the dark/box maze did not significantly worsen during disease progression, nor were these parameters modified in fingolimod treated EAE mice. Further studies are required to better define these aspects.

In recent years, evidence has shown that release efficiency at glutamatergic and GABAergic nerve terminals isolated from the cortex and the spinal cord of EAE mice is modified when compared to control [8]. In particular, glutamate exocytosis from cortical nerve endings of EAE mice was significantly reduced starting from the early, almost asymptomatic, stage of disease (14 d.p.i.) and it did not recover during and after the acute stage of disease. Differently, glutamate outflow from spinal cord terminals was unmodified at the early stage of disease, but it increased dramatically during and after the acute stage of disease. The first finding of the present study is that *in vivo* prophylactic fingolimod efficiently restored the above-mentioned presynaptic impairments in EAE mice. The efficiency of glutamate and GABA exocytosis efficiencies in the cortex and in the spinal cord of EAE mice prophylactically administered with fingolimod dissolved in the drinking water was significantly different to that observed in untreated EAE mice, but largely comparable to those levels observed in control, non-immunized, mice.

The mechanism(s) accounting for these beneficial effects should therefore be charcaterized. The possibility that fingolimod modulates *directly* chemical transmission by acting presynaptically at active synapses in both animals and patients suffering from demyelinating disorders has been already proposed [16, 22] and, undoubtedly, deserves further attention [37]. Actually, S1PRs (the preferential targets of the fingolimod-phosphate, the active form of the drug) exist at the presynaptic component of chemical synapses, where their activation modulates glutamate release [38–40] by controlling the assembly of the Soluble NSF Attachment Protein receptor (SNARE) complex [41]. Inasmuch, in a very recent paper, Riganti et al. [42] demonstrated that S1PRs also control synapsin I relocation, thus promoting glutamate exocytosis. Taking into account our recent data demonstrating that presynaptic adaptive modifications elicited by *in vivo* drug administration (*i*.*e*., fingolimod) are retained by isolated nerve endings and could be highlighted in *ex vivo* and *in vitro* studies as changes in transmitter release efficiency [9,11,43]. The possibility that *in vivo* fingolimod had triggered *direct* and persistent beneficial adaptations at nerve terminals that could be quantified in *ex-vivo* and *in vitro* experiments seems plausible. However, the following observations contrast this conclusion: i) *in vivo* oral fingolimod did not affected *per se* the spontaneous and the evoked release of glutamate or GABA in control mice, and ii), the fingolimod-evoked reduction of glutamate exocytosis in spinal cord terminals seems inconsistent with the SPR1-mediated facilitation of SNARE assembly reported to occur in neurones. Actually, in neurones, activation of neuronal S1PRs was associated to increased SNARE association, the inhibition of SNARE assembly being an event so far described to preferentially occur in astrocytes [39,44,45].

A more conservative hypothesis is that, in addition to the *direct* effects at glutamatergic nerve terminals, some *indirect* mechanisms (*i*.*e*., the modulation of pathological processes that take place behind the onset of synaptic alterations, see Rossi et al., [21]) could concur to determine the beneficial effects observed in fingolimod administered EAE mice. As to the *indirect* effect(s), i*n vivo* chronic fingolimod influences, at various levels, the inflammatory pathway(s) accounting for disease progression. In particular, the drug regulates the migration of T-cells and the production of cytokines in the CNS; these two events prime disease etiopathogenesis, but also modulate central glutamate and GABA transmission [4,46–48]. Actually, circulating T-cells as well as cytokines/chemokines control glutamate transmission in EAE mice, possibly by acting presynaptically at chemical synapses [4,49]. By reducing these immune-mediated events, fingolimod can delay the course of the disease [50], also slowing the onset of synaptic defects at glutamatergic synapsis. Of note, pro-inflammatory chemokines such as CCL5 and CXCL12 presynaptically control glutamate exocytosis in healthy conditions, either facilitating or inhibiting this event [30,49,51]. In particular, CCL5 inhibits glutamate exocytosis from cortical synaptosomes, but it facilitates glutamate overflow from spinal cord terminals. In a previous work, the CCL5 overexpression observed starting from the early stage of demyelinating disorder was proposed to account for the altered glutamate exocytosis detected in cortical and spinal cord glutamate nerve endings of EAE mice [9]. Accordingly, drug-induced reduced CCL5 overexpression in the CNS of EAE mice led to a significant restoration of glutamate exocytosis from nerve terminals Interestingly, fingolimod normalizes CCL5 overexpression in pathological conditions [52], an event also shown in the present data that might account for the beneficial effects that occur following its oral administration.

In addition to the antiinflammatory action that is beneficial to central transmission, fingolimod also can *indirectly* modulate transmitter release at nerve terminals by acting at astrocytes and microglial cells neighbouring synaptic processes. Actually, astrocytes influence glutamate availability in the biophase. It has been proposed that excessive glutamate release from astrocytes can alter the functions of adjacent neurones [53,54]. Inasmuch, microglia control glutamate availability in the synaptic cleft by acting as cell scavenger of excessive glutamate [4]. By acting at glial S1PRs, *in vivo* fingolimod should prevent the pathological astrocyte to neuron signalling [39,44,45], then restoring physiological conditions. Accordingly, in the present results, fingolimod prevents both microglia and astrocyte activation.

Whatever the mechanism, the restoration of glutamate exocytosis elicited by *in vivo* fingolimod might have a role in the amelioration of clinical symptoms and altered emotional behaviour in EAE mice, as indeed observed. Actually, reduced glutamate release efficiency in the cortex and in the hippocampus has been correlated to stress-related disorders [55,56], and emotional symptoms become more severe as the disease progresses [27,57,58]. Inasmuch, impaired glutamate and GABA transmission in the spinal cord correlates with nociceptive sensitivity [59]. Recent observations have shown that most of these EAE-associated symptoms were significantly reduced in animals administered with lower doses of fingolimod [36]. A significant amelioration of clinical score and emotional behaviour was highlighted in studies aimed at comparing the impact of prophylactic versus therapeutic fingolimod treatments in EAE mice [16,24,36,60]. Accordingly, we provided evidence that prophylactic fingolimod administered at lower doses (0.1–0.03 mg/kg) efficiently recovered the presynaptic cortical defects in EAE mice. When therapeutically administered in ongoing disease (*i*.*e*., starting from the acute stage of the disease), fingolimod-mediated restoration of transmitter release was detectable in the cortex and the hippocampus, but not in the spinal cord nerve terminals, where glutamate exocytosis was unmodified when compared to untreated EAE mice. Very interestingly, the experiments aimed at delucidating the effect of therapeutic fingolimod allowed to highlight the late impairment in glutamate transmission in the hippocampus. These hippocampal defects could account for the mood disorders and the loss of memory often observed in MS patients. Further studies are required to better define the role of these alterations.

The lack of efficacy of fingolimod to recover pathological glutamate exocytosis at the spinal cord level when the drug is applied at lower dose or following a therapeutic protocol deserves some comments. Evidence in the literature suggests that the gravity of the inflammation and demyelination is less pronounced in the cortex when compared to that observed in the spinal (missing a noun), at least during and immediately after the acute stage of disease [4,61–66]. If this is the case, it is possible that the compensatory mechanisms triggered by low and/or therapeutic fingolimod to rescue presynaptic defects in the spinal cord are insufficient (or even exhausted), at least when compared to those occurring in the cortex and the hippocampus. Further investigation however is required to give a rationale for these discrepancies.

### Conclusions

Our study supports the notion that altered release efficiency occurs at glutamatergic and GABAergic nerve terminals in the CNS of EAE at different stages of disease. Release impairments became detectable starting from the acute stage of the disease in the cortex and the spinal cord and, after the acute stage, in the hippocampus. This impairment may have a role in determining cognitive and motor deficits as well as mood disorders, which represent common symptoms of disease progression. *In vivo* fingolimod prevents these defects to a different extent, in a dose- and a region-dependent manner. The drug is extremely potent when administered prophylactically, starting from the acute stages of the disease, but becomes less efficient when administered therapeutically. In the latter case, the drug exerts its beneficial effects in the cortex and in the hippocampus, but not in the spinal cord of EAE mice, where glutamate and GABA defects remain clearly evident. Although caution must be taken when extrapolating mouse studies to humans [*i*.*e*., the EAE mice model has been generally used to study the spinal cord impairments in MS, although recent data led to propose it as a suitable model to investigate also brain impairments, 62–66], our findings suggest that prophylactic intervention strategy with fingolimod might reduce the gravity of neuronal defects in the progressive form of MS. This conclusion is particular relevant when considering that: i) fingolimod is a proposed therapy for the relapsing-remitting form of MS, and ii) the recent technological advances in *in vivo* non-invasive studies makes it possible the discovery of pharmacological prophylactic regimens for this disease. Although further studies are required to define exactly the molecular events accounting for the restoration of glutamate and GABA exocytosis, the possibility that fingolimod has primed nerve terminals by directly acting at presynaptic S1PRs seems consistent with the restoration of glutamate exocytosis in the cortex and in the hippocampus of EAE mice. However, at the spinal cord level, fingolimod-mediated indirect effects relying on the anti-inflammatory activity of the drug (*i*.*e*., the reduced expression of CCL5) might better account for the restoration of presynaptic functions observed following fingolimod administration.
