# Correction: Prophylactic versus Therapeutic Fingolimod: Restoration of Presynaptic Defects in Mice Suffering from Experimental Autoimmune Encephalomyelitis

**DOI:** 10.1371/journal.pone.0292584

**Published:** 2023-10-03

**Authors:** Tommaso Bonfiglio, Guendalina Olivero, Elisa Merega, Silvia Di Prisco, Cristina Padolecchia, Massimo Grilli, Marco Milanese, Lorenzo Di Cesare Mannelli, Carla Ghelardini, Giambattista Bonanno, Mario Marchi, Anna Pittaluga

After this article [1] was published, concerns were raised about some of the microscopy panels in Figs 7 and 9. Specifically:

- The control panel is duplicated as the control + fingolimod panel in Fig 7A.
- In the EAE panel in Fig 9B, the lower right quadrant is discontinuous with the rest of the image, and this region is at a higher magnification than the rest of the image.

In response to queries about the experiments in Fig 7A, the authors stated that the control panel in Fig 7A was duplicated as the control + fingolimod panel in Fig 7A in error. An updated version of Fig 7 with the correct control + fingolimod panel in Fig 7A from the time of the original experiments is provided here. The original data underlying Fig 7 can be found in S1 File.

**Fig 7:**
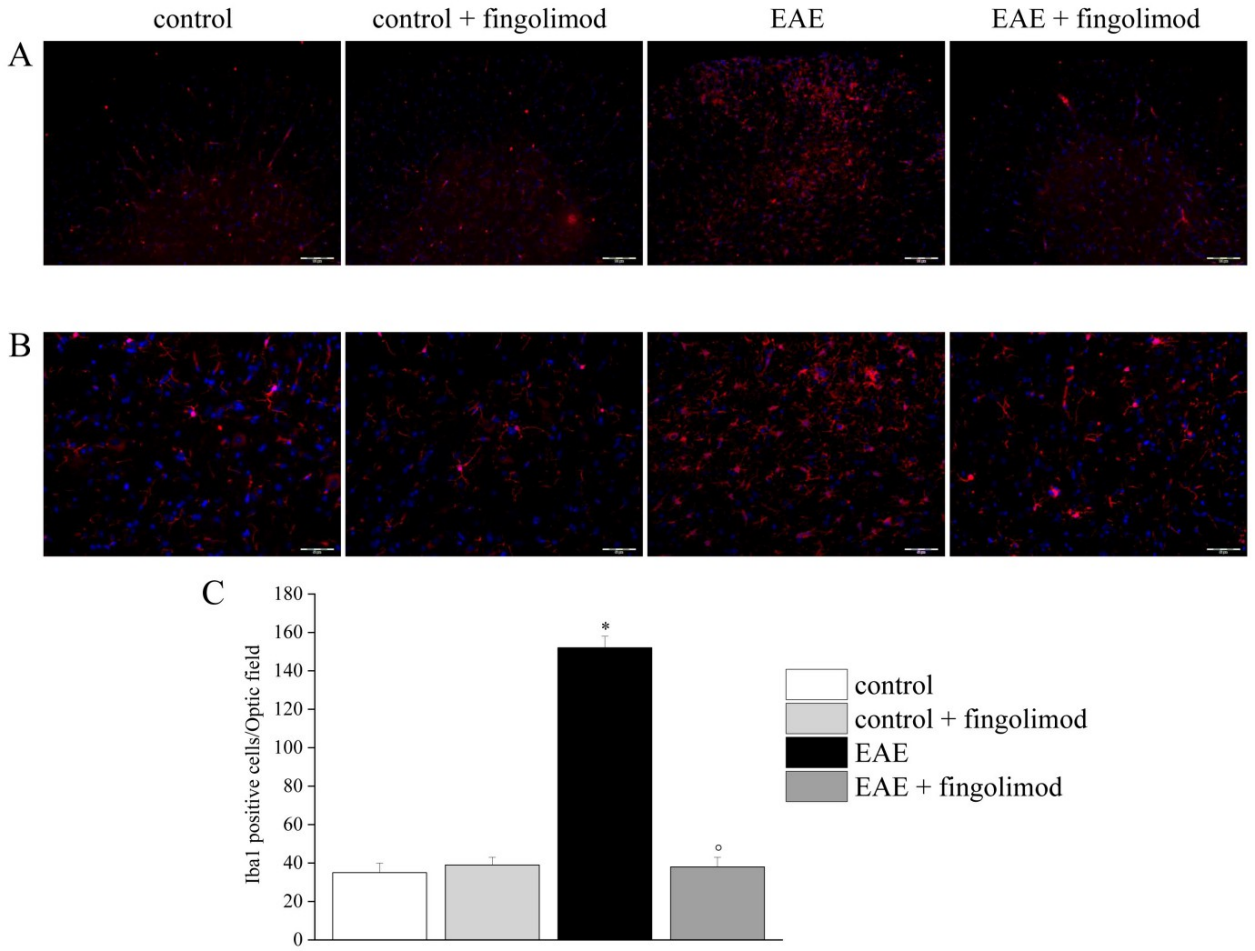
Effects of *in vivo* prophylactic fingolimod on microglial cells in the spinal cord of EAE mice at the acute stage of disease. On day 21 post EAE induction, tissue sections were immuno-stained with the anti-Iba1 antibody (red) to recognize microglia cells and with DAPI (blue) to identify cell nuclei. (**A**) 10X: Low-magnification image of spinal cord sections. (**B**) 20X: High-magnification image of the spinal cord sections. (**C**) Quantitative evaluation of the number of Iba1-positive cells/Optic field in the spinal cord of mice of each treatment-group. * p < 0.05 *versus* all other groups; ° p < 0.05 versus untreated EAE mice.

In response to queries about the experiments in Fig 9B, the authors stated that the EAE panel in shows an insert with higher magnification (40x) to show the alterations induced by EAE. Updated versions of Fig 9 with the insert in the EAE panel in Fig 9B, and its caption, are provided here. The original data underlying Fig 9 can be found in S1 File.

**Fig 9:**
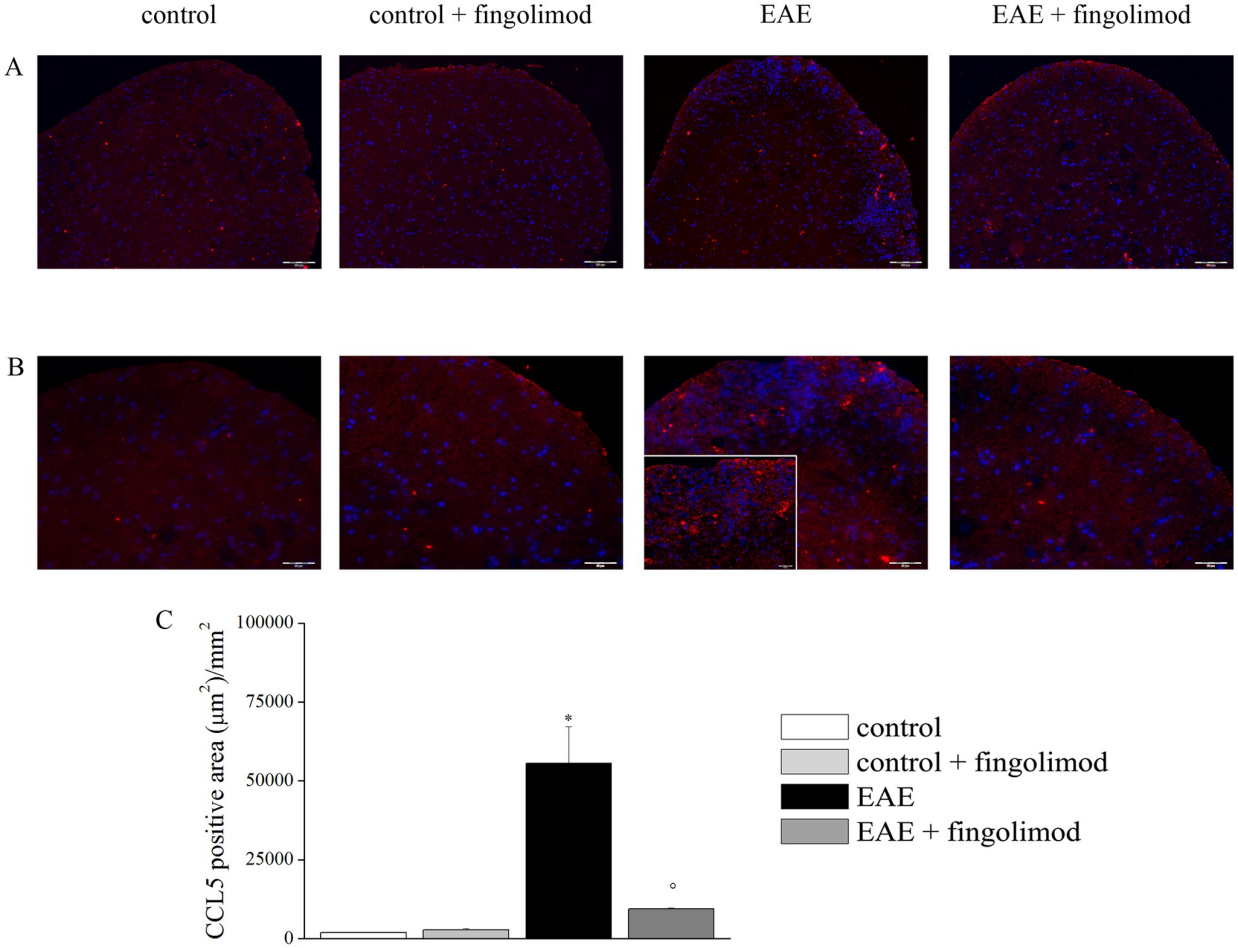
Effects of *in vivo* prophylactic fingolimod on CCL5 in the spinal cord of EAE mice at the acute stage of disease. On day 21 post EAE induction, tissue sections were immuno-stained with the anti-CCL5 antibody (red) and with DAPI (blue) to identify cell nuclei. (**A**) 10X: Low-magnification image of spinal cord sections; (**B**) 20X: High-magnification image of the spinal cord sections. The EAE panel contains an insert with 40X magnification.; (**C**) The CCL5 positive area (μm^2^) /mm^2^ in the spinal cord of mice of each treatment-group is reported. * p < 0.05 *versus* control untreated mice; ° p < 0.05 *versus* untreated EAE mice.

The original data underlying the control and control + fingolimod panels in Fig 7A and the EAE panel in Fig 9B have been reviewed by PLOS. The remainder of the data underlying article [1] are available from the authors.

The authors apologize for the errors in the published article.

## Supporting information

S1 File Original underlying images for the panels in Figs 7A, 7B, 9A and 9B, and individual-level underlying data for Figs 7C and 9C. (ZIP)
