# Supplementary figures and images for: Correction: Prophylactic versus Therapeutic Fingolimod: Restoration of Presynaptic Defects in Mice Suffering from Experimental Autoimmune Encephalomyelitis
Source: PLoS One. 2023 Oct 3;18(10):e0292584. doi: 10.1371/journal.pone.0292584 (PMC10547186; doi:10.1371/journal.pone.0292584)

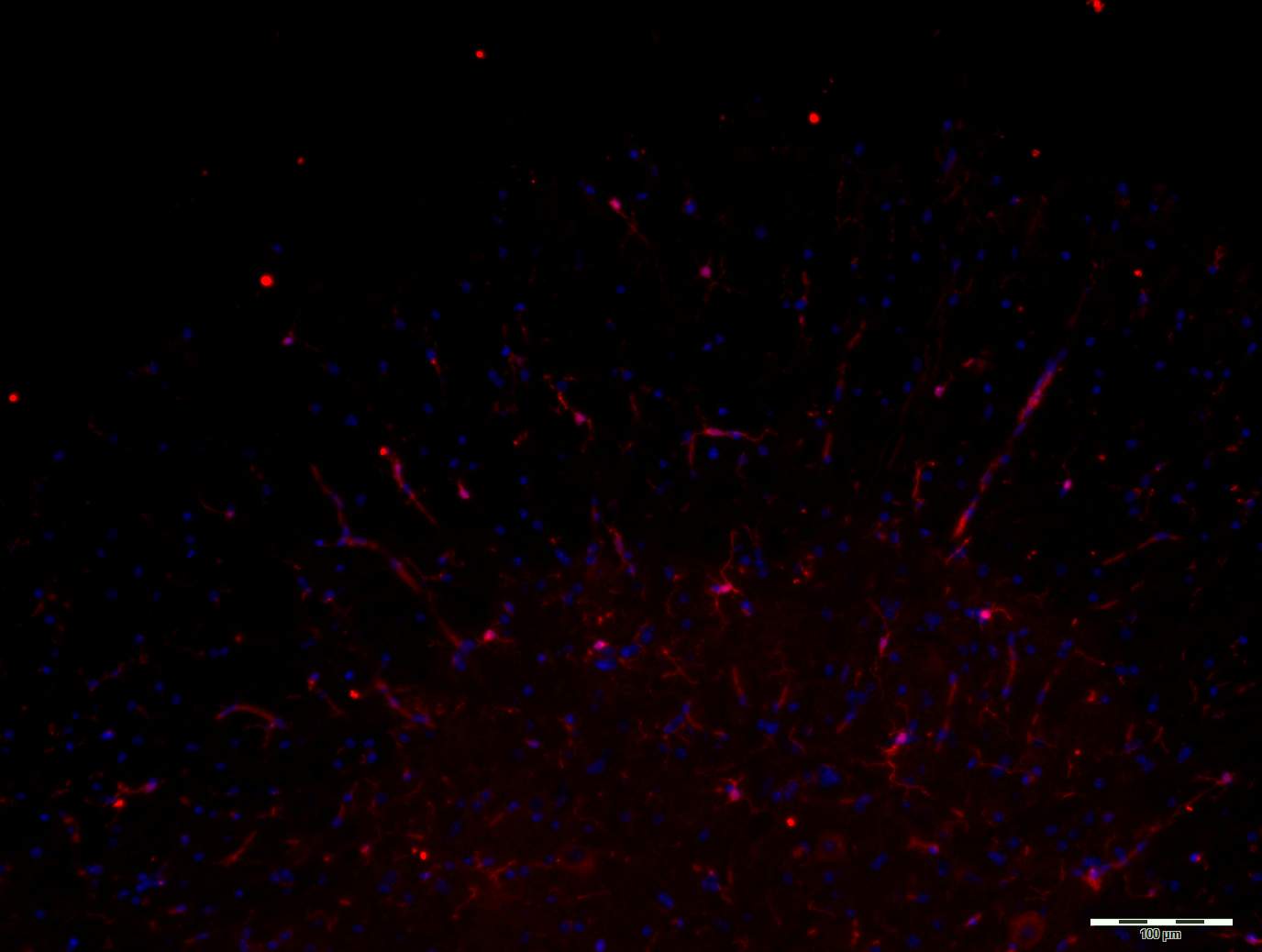

Supplement: S1 File — (ZIP) [file pone.0292584.s001.zip › pone0170825 files/Figure 7/CTR funicolo ant 10x.tif]

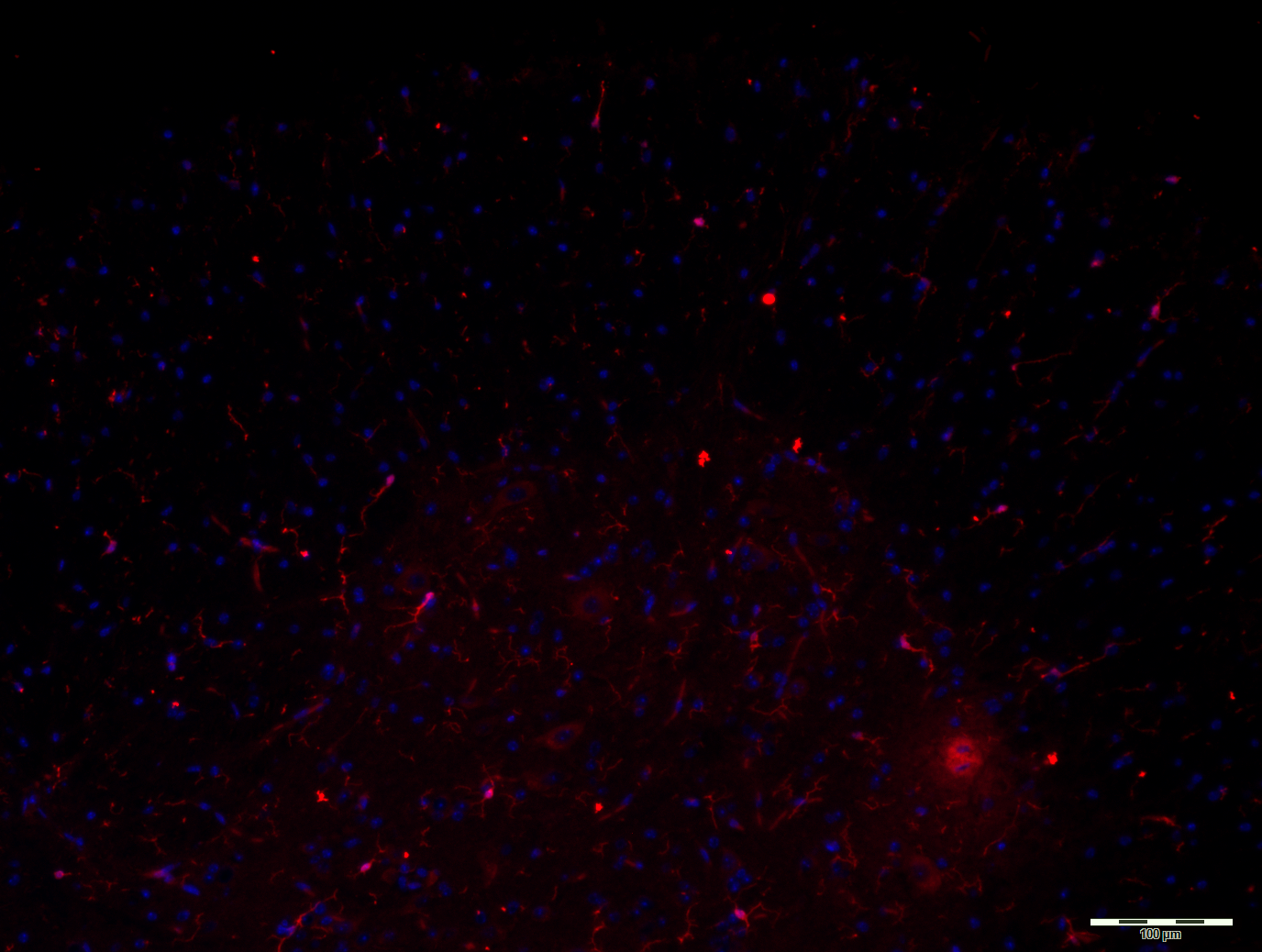

Supplement: S1 File — (ZIP) [file pone.0292584.s001.zip › pone0170825 files/Figure 7/CTR+FTY funicolo anteriore 10x B.tif]

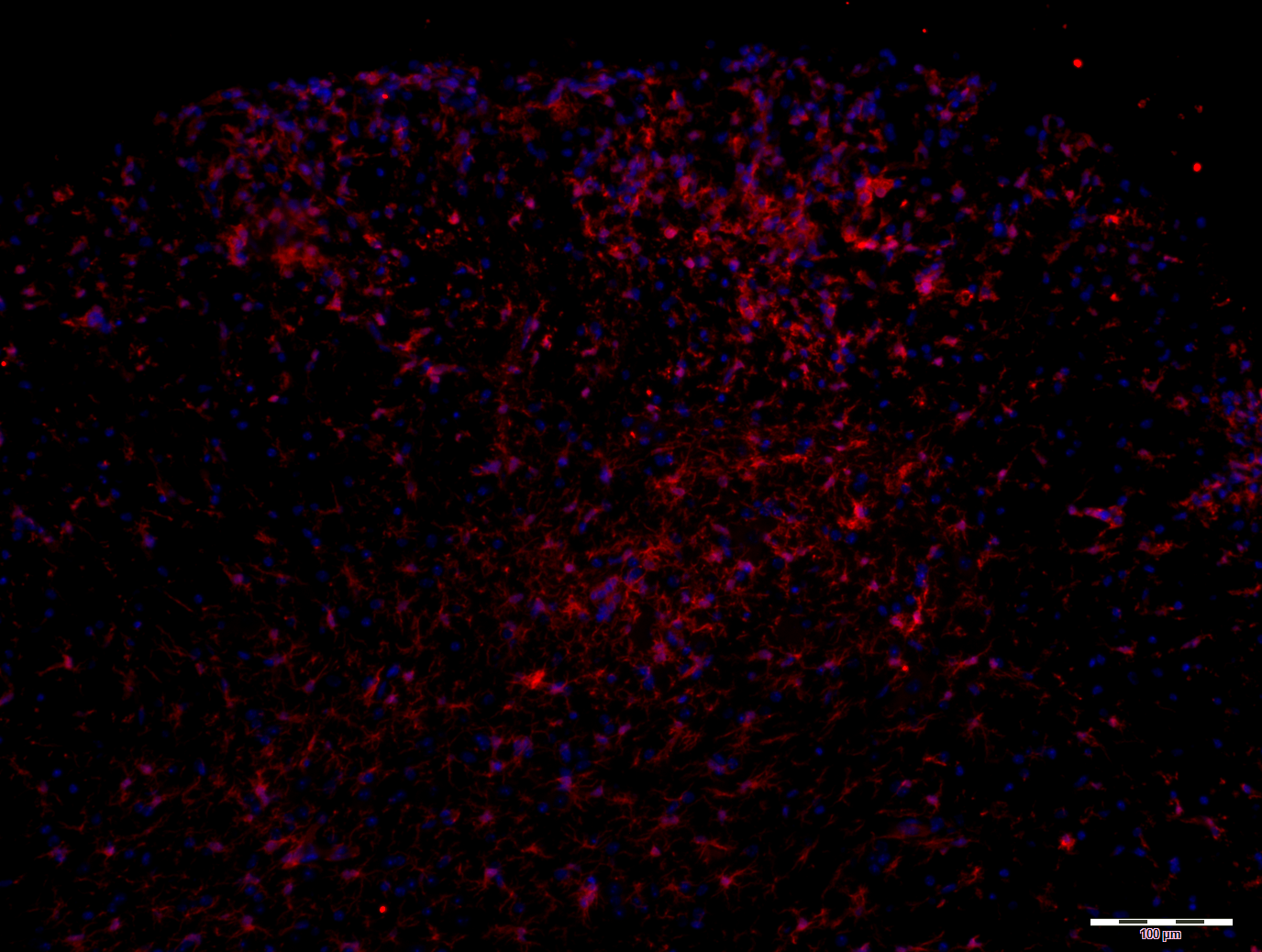

Supplement: S1 File — (ZIP) [file pone.0292584.s001.zip › pone0170825 files/Figure 7/EAE funicolo ant 10x.tif]

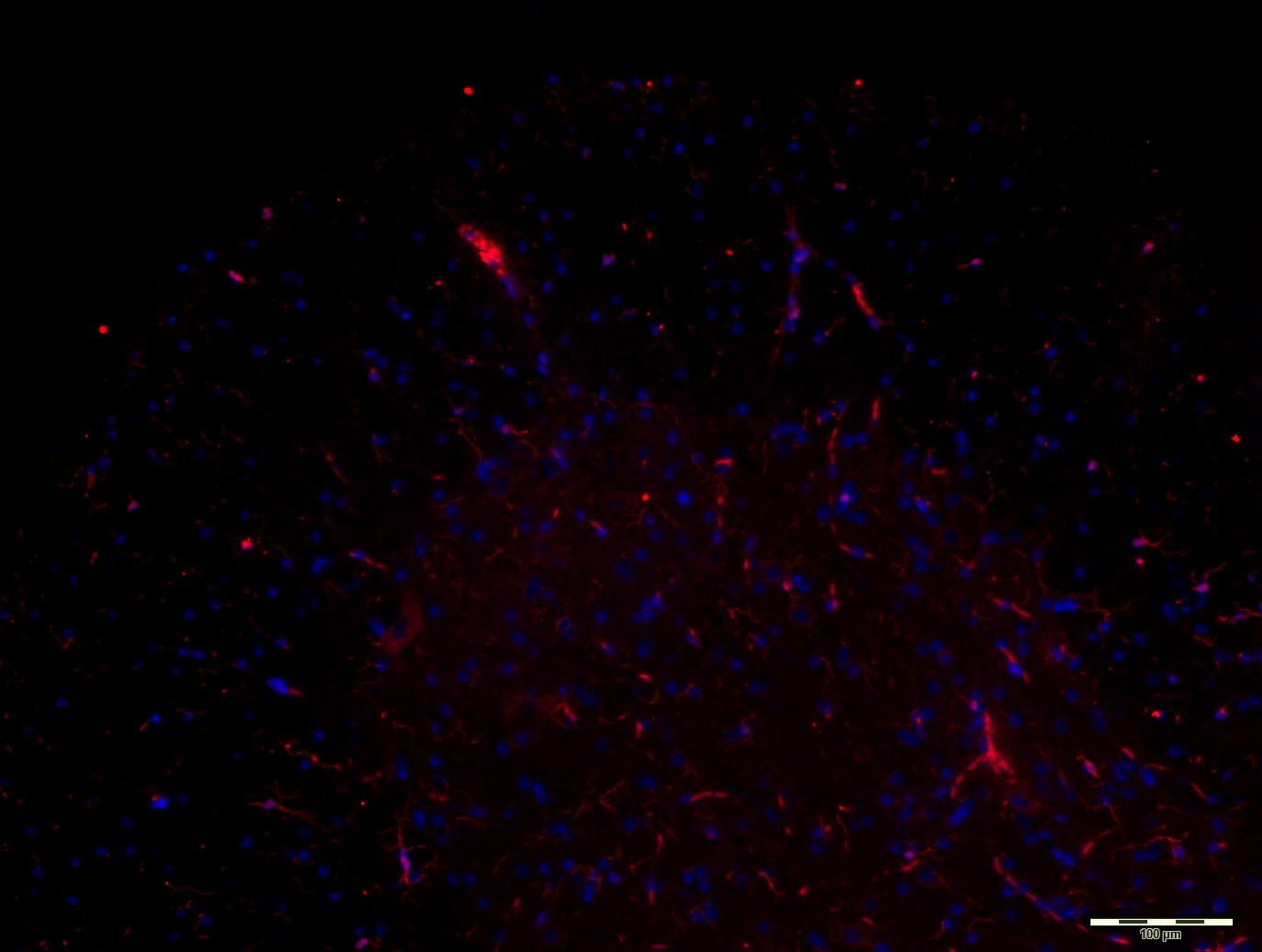

Supplement: S1 File — (ZIP) [file pone.0292584.s001.zip › pone0170825 files/Figure 7/EAE+FTY funic anteriore 10x.tif]

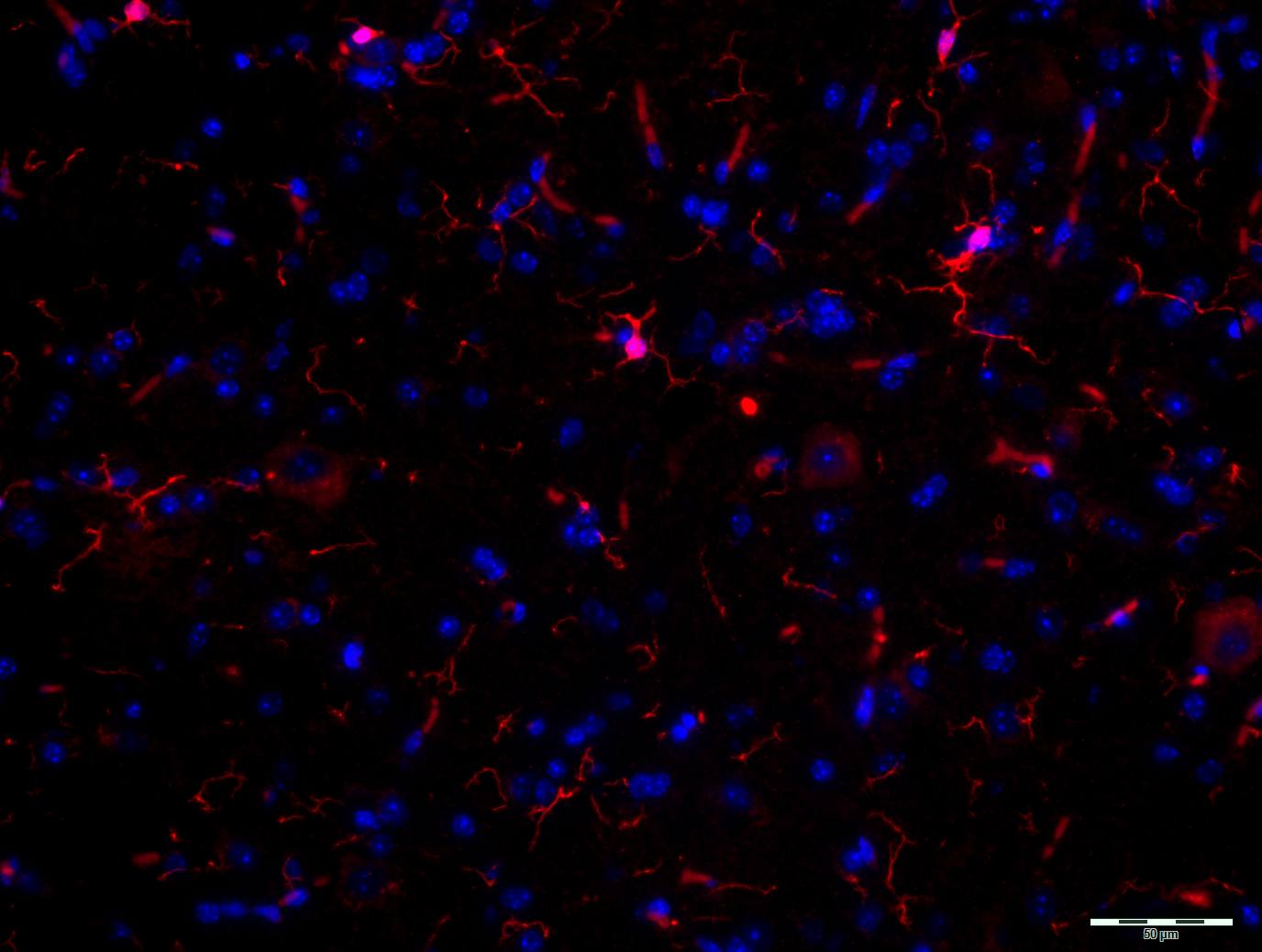

Supplement: S1 File — (ZIP) [file pone.0292584.s001.zip › pone0170825 files/Figure 7/iba1 CTR sost grigia 20x.tif]

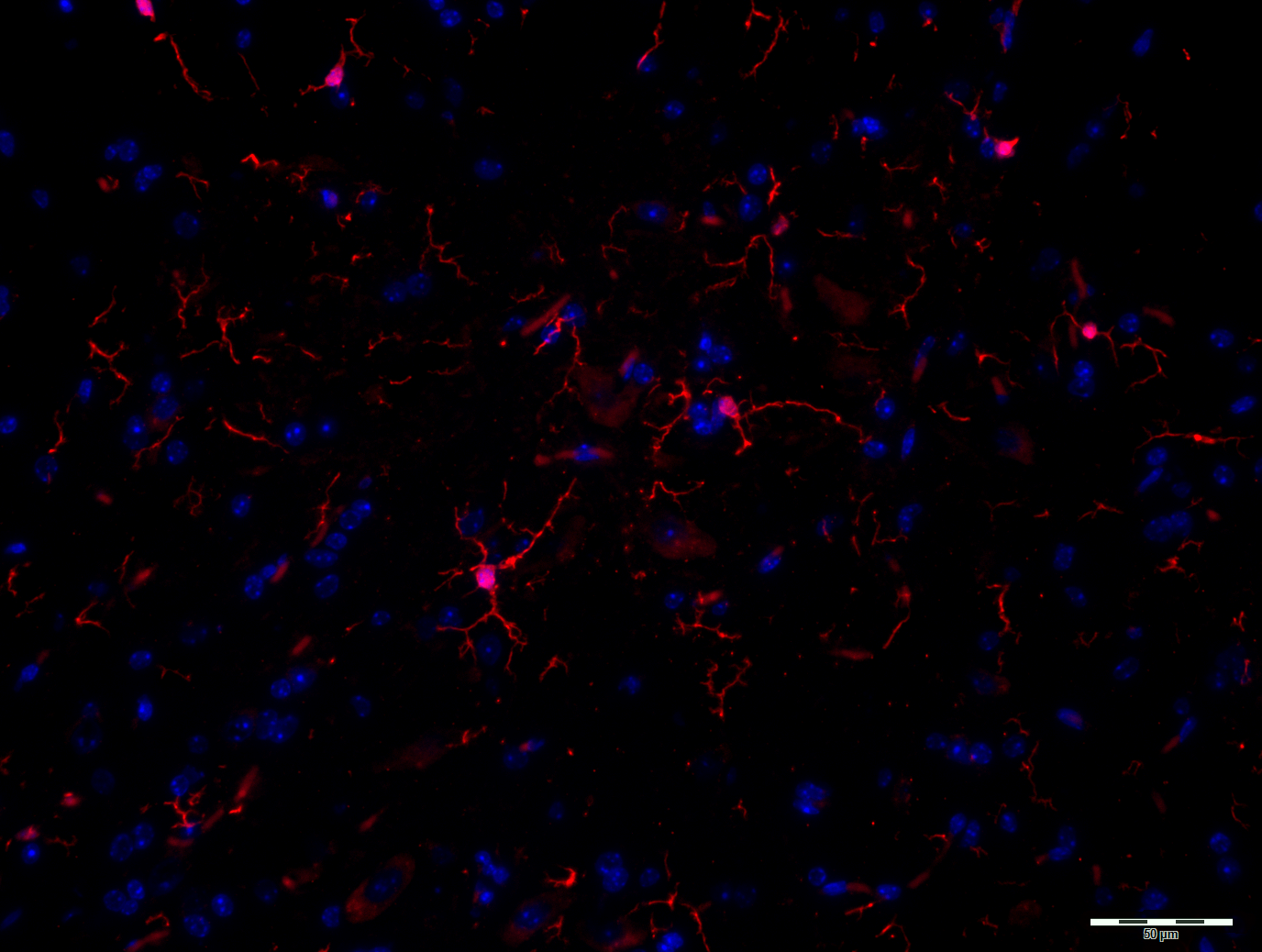

Supplement: S1 File — (ZIP) [file pone.0292584.s001.zip › pone0170825 files/Figure 7/iba1 CTR+FTY720 sost grigia 20x.tif]

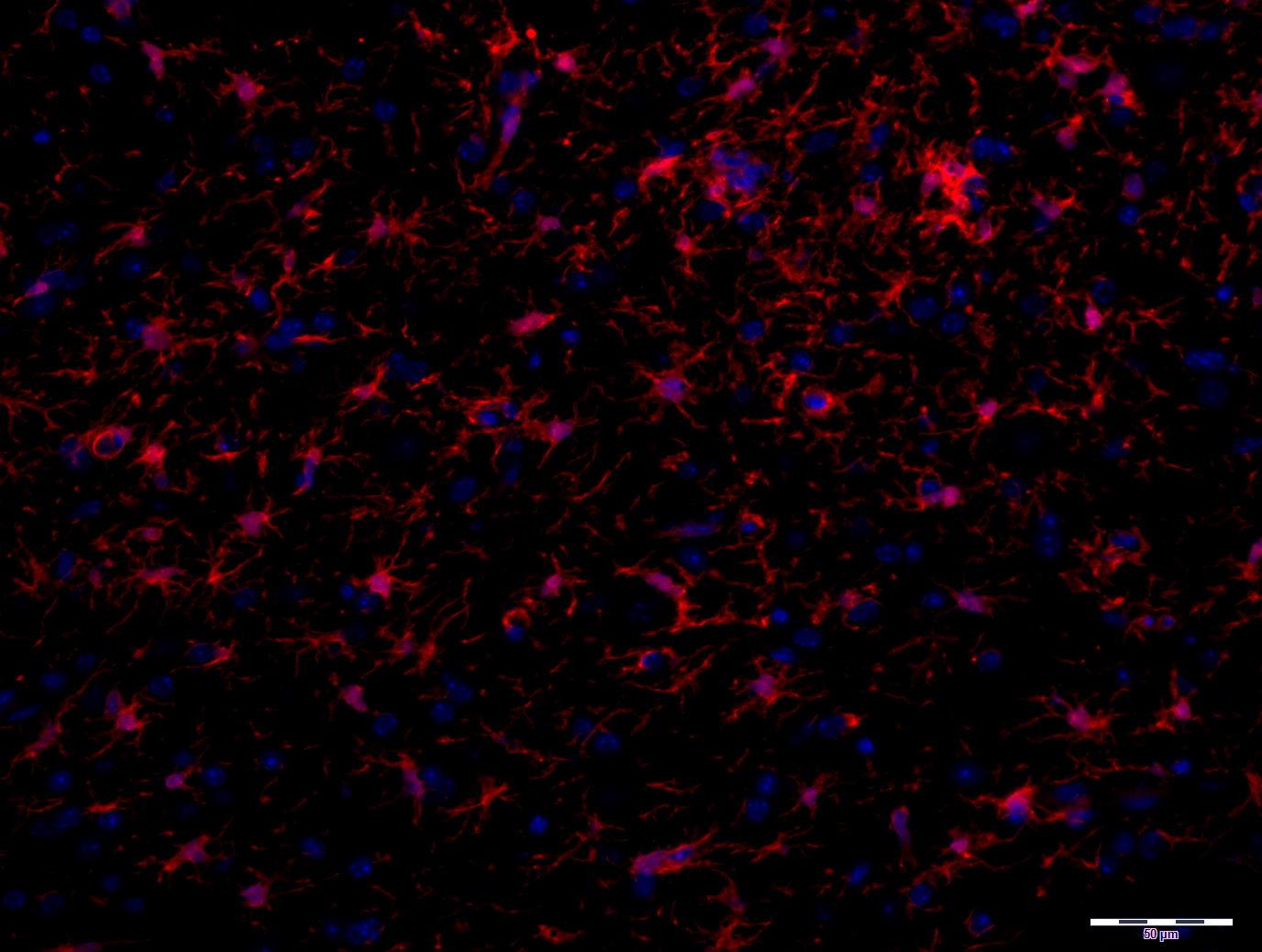

Supplement: S1 File — (ZIP) [file pone.0292584.s001.zip › pone0170825 files/Figure 7/iba1 EAE sost grigia 20x.tif]

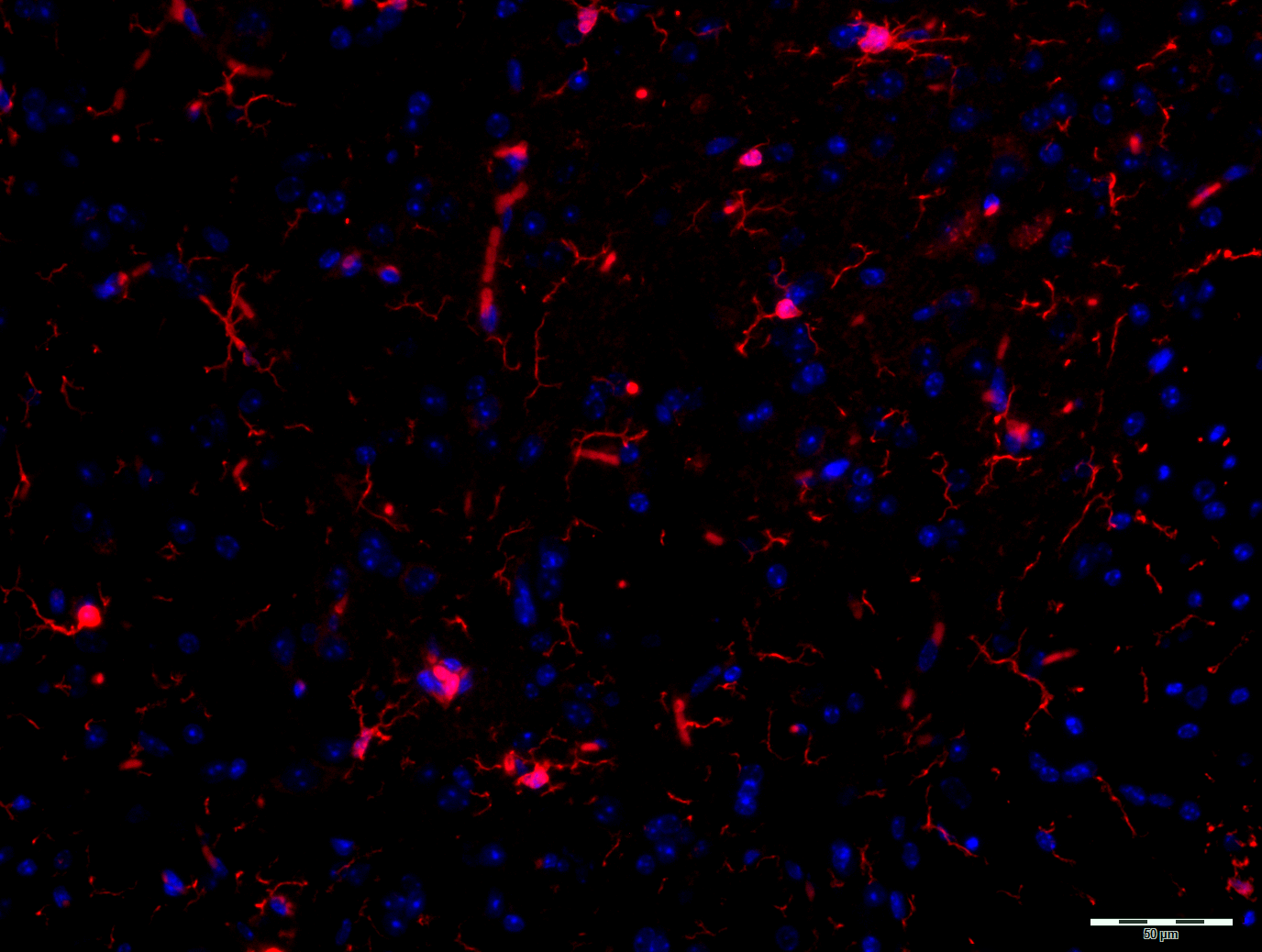

Supplement: S1 File — (ZIP) [file pone.0292584.s001.zip › pone0170825 files/Figure 7/iba1 EAE+FTY sost grigia 20x B.tif]

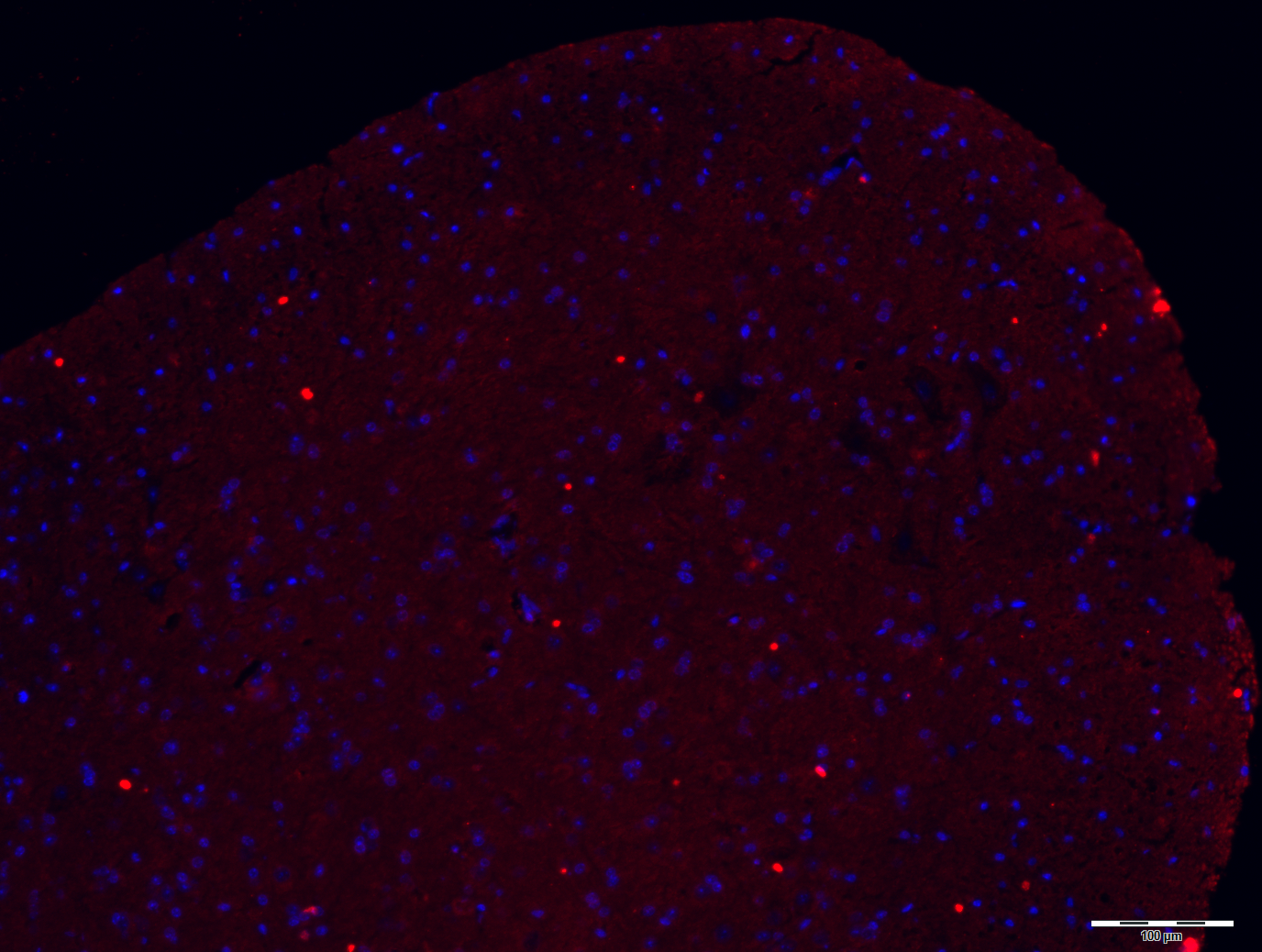

Supplement: S1 File — (ZIP) [file pone.0292584.s001.zip › pone0170825 files/Figure 9/RANTES_ctr_corno ant_10x.tif]

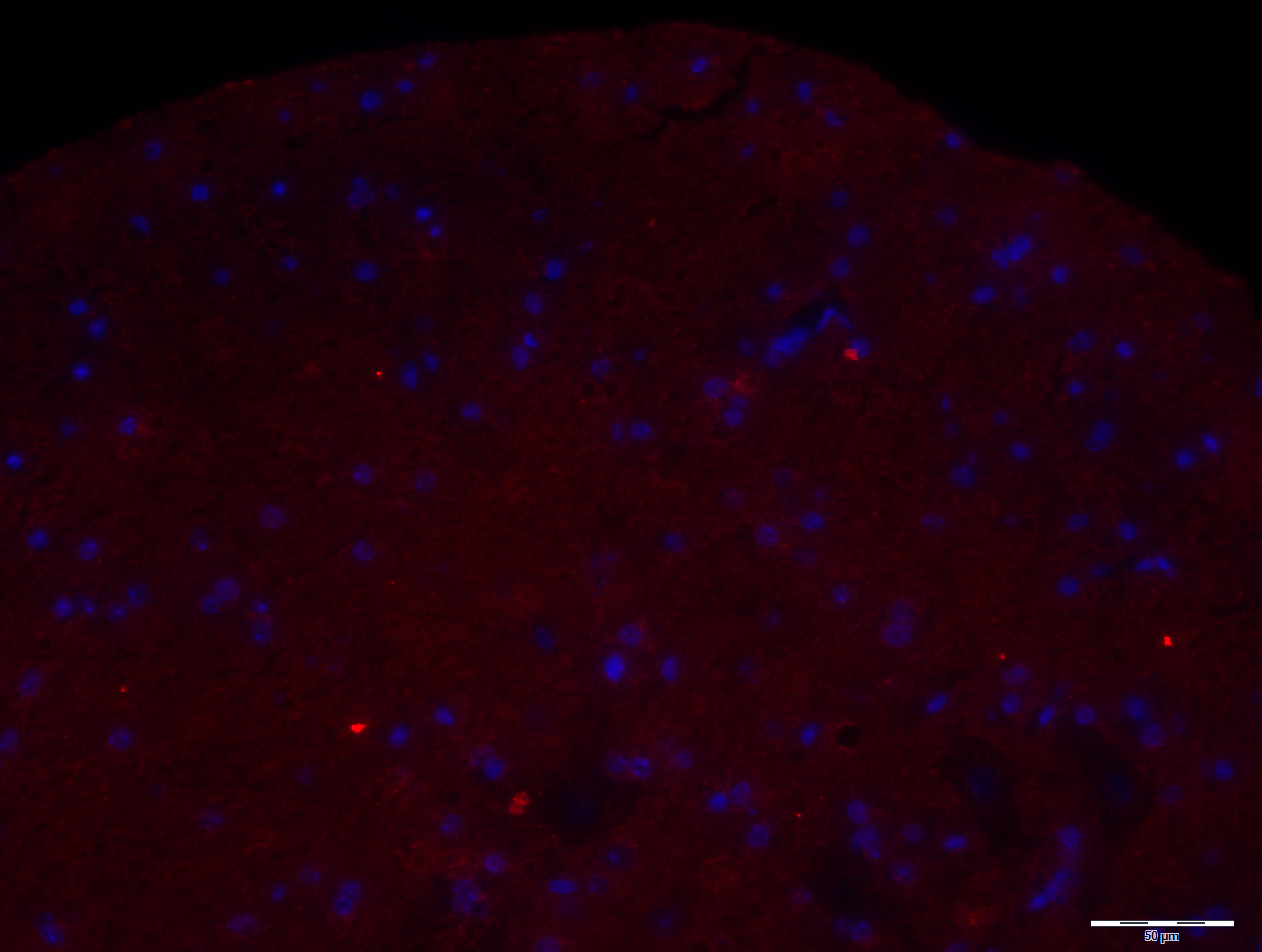

Supplement: S1 File — (ZIP) [file pone.0292584.s001.zip › pone0170825 files/Figure 9/RANTES_ctr_corno ant_20x.tif]

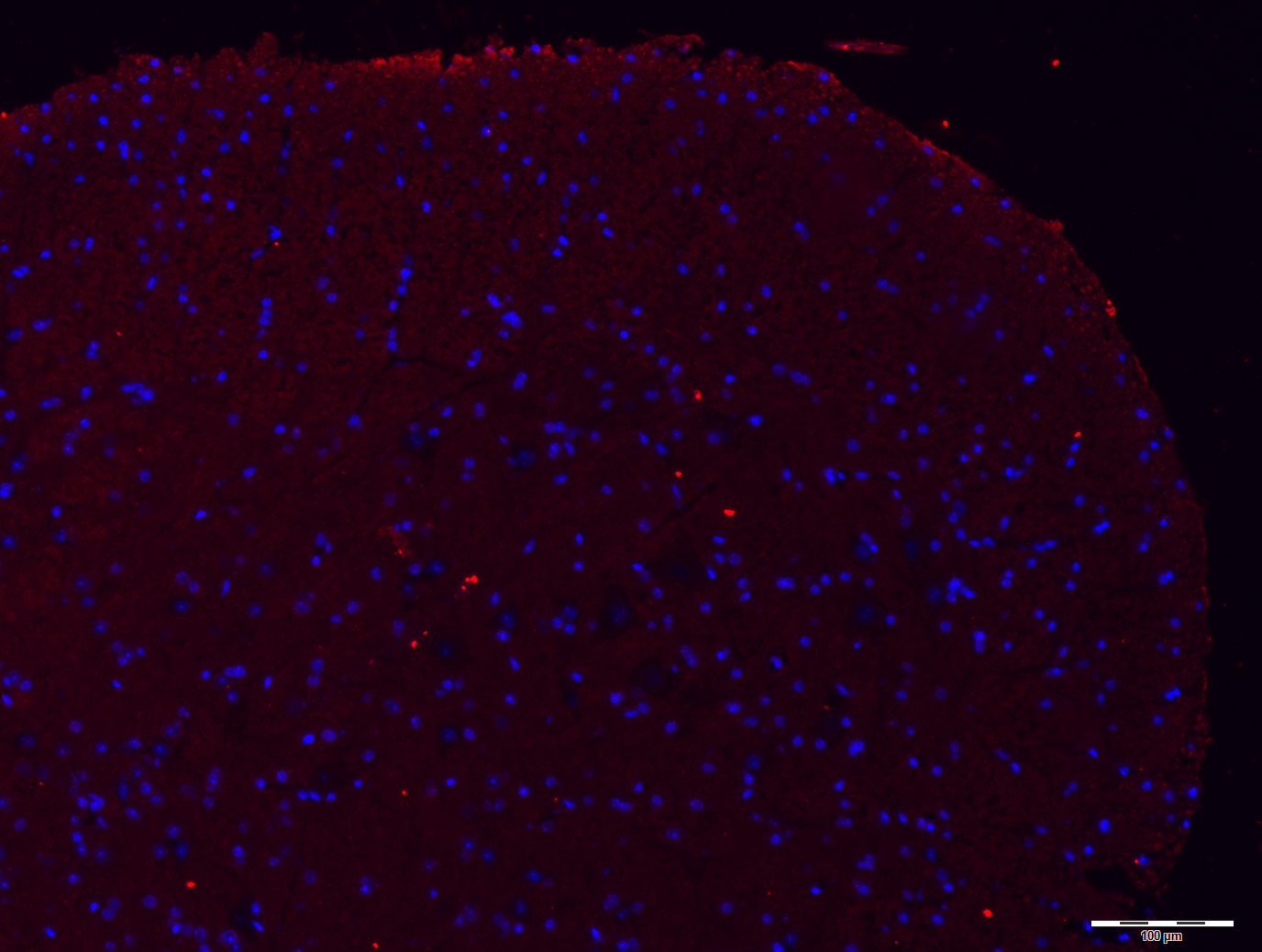

Supplement: S1 File — (ZIP) [file pone.0292584.s001.zip › pone0170825 files/Figure 9/RANTES_ctr+fingo_corno ant_10x.tif]

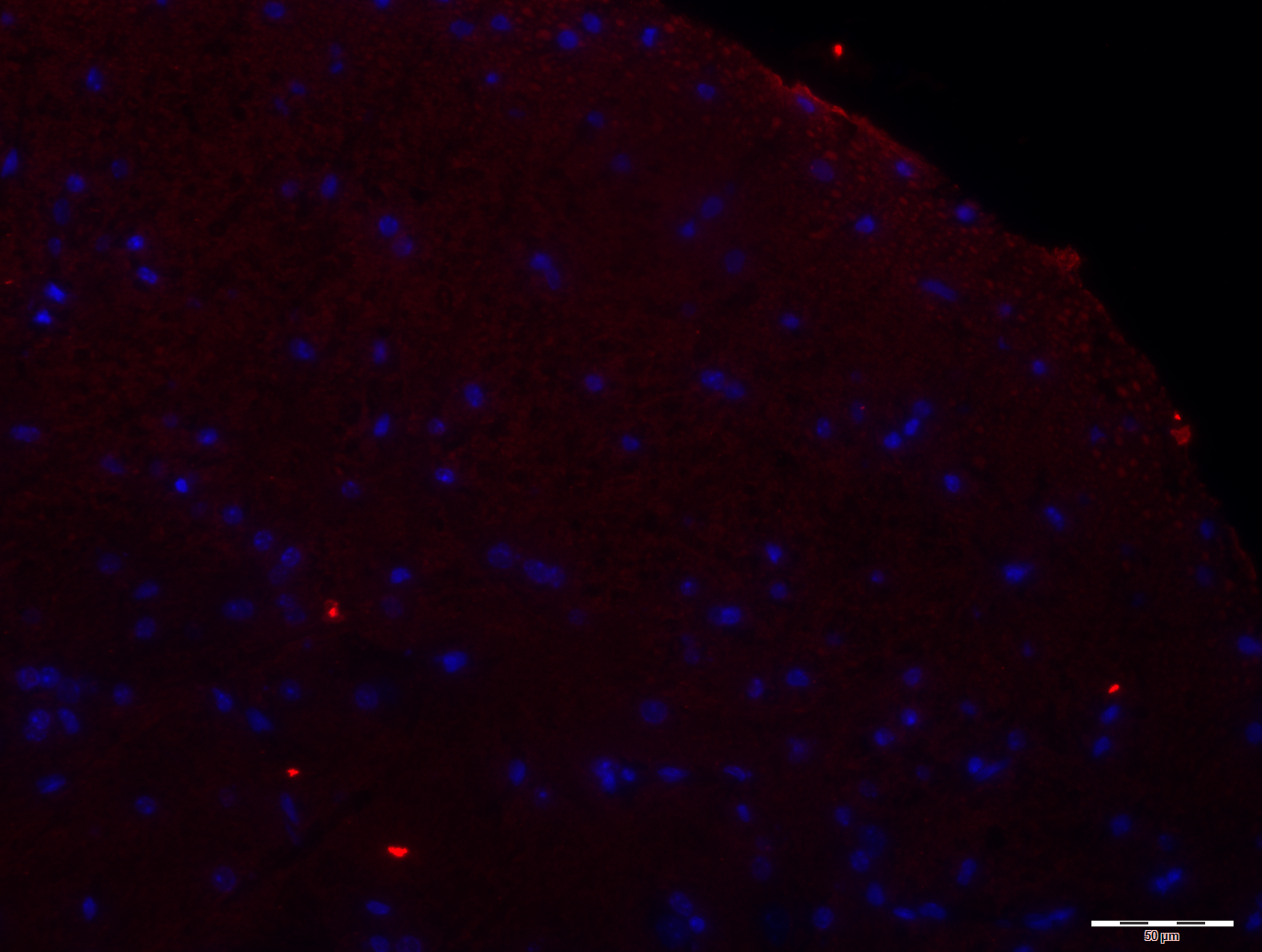

Supplement: S1 File — (ZIP) [file pone.0292584.s001.zip › pone0170825 files/Figure 9/RANTES_ctr+fingo_corno ant_20x.tif]

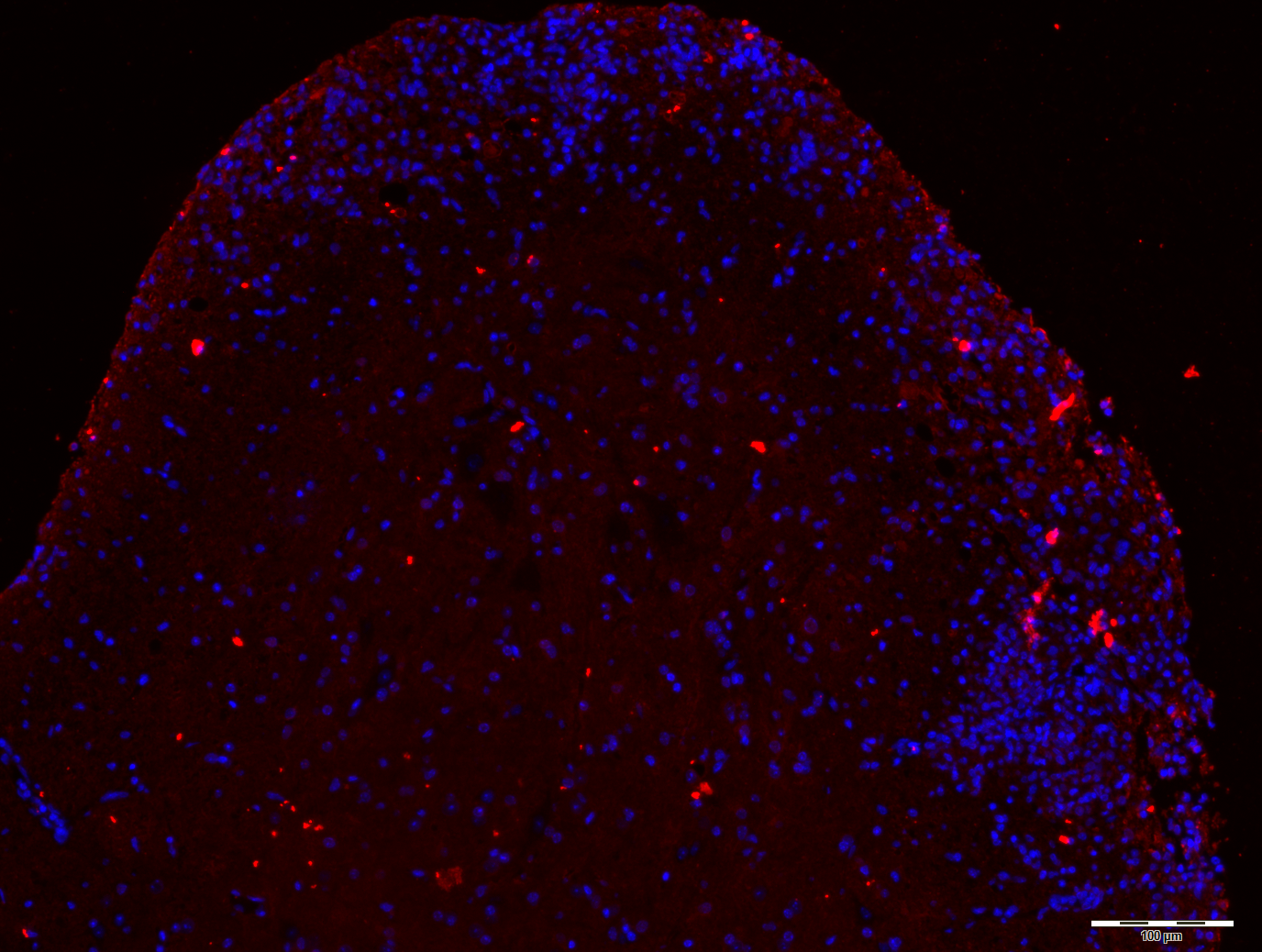

Supplement: S1 File — (ZIP) [file pone.0292584.s001.zip › pone0170825 files/Figure 9/RANTES_EAE_corno ant_10x.tif]

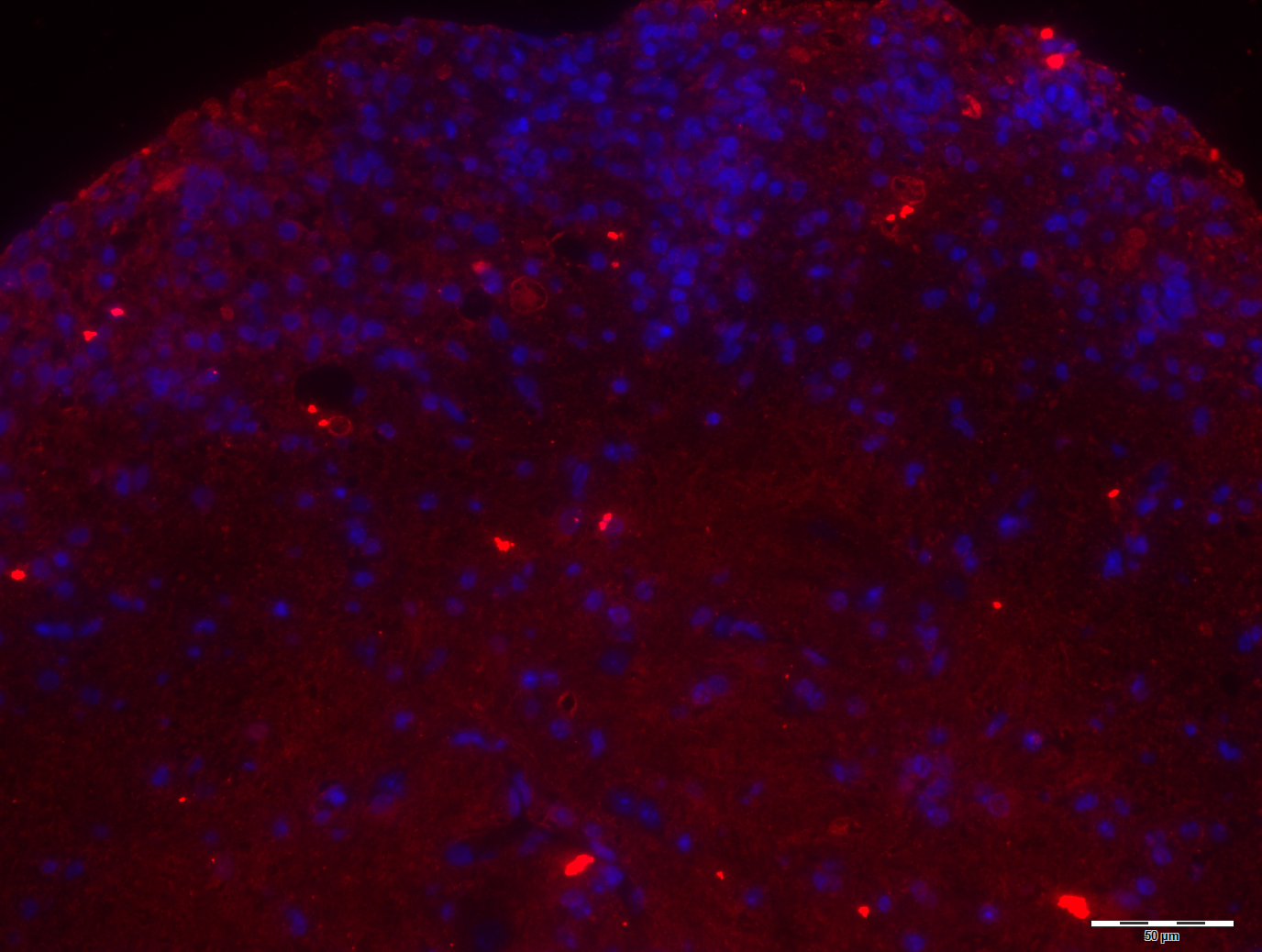

Supplement: S1 File — (ZIP) [file pone.0292584.s001.zip › pone0170825 files/Figure 9/RANTES_EAE_corno ant_20x 3.tif]

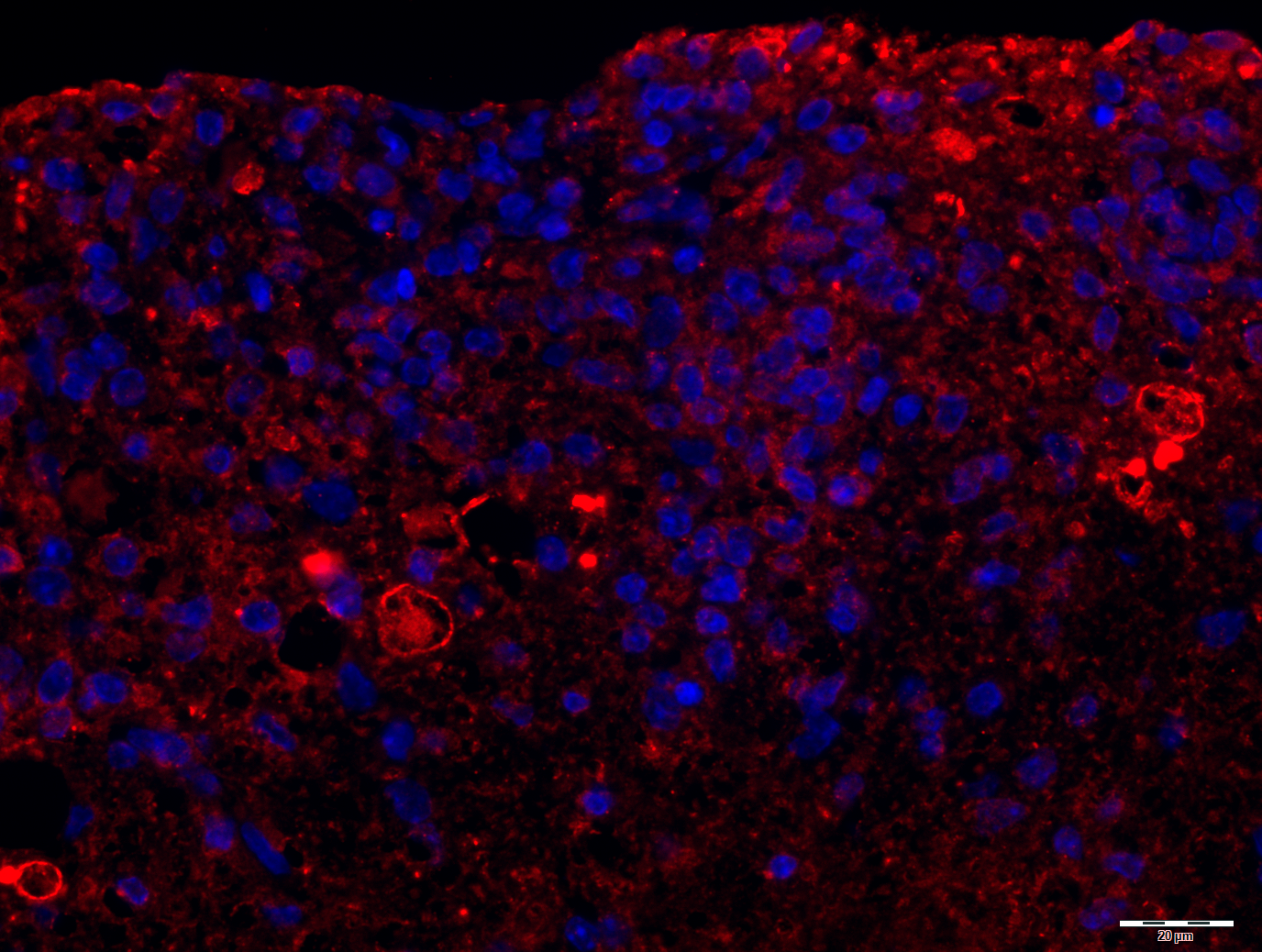

Supplement: S1 File — (ZIP) [file pone.0292584.s001.zip › pone0170825 files/Figure 9/RANTES_EAE_corno ant_40x.tif]

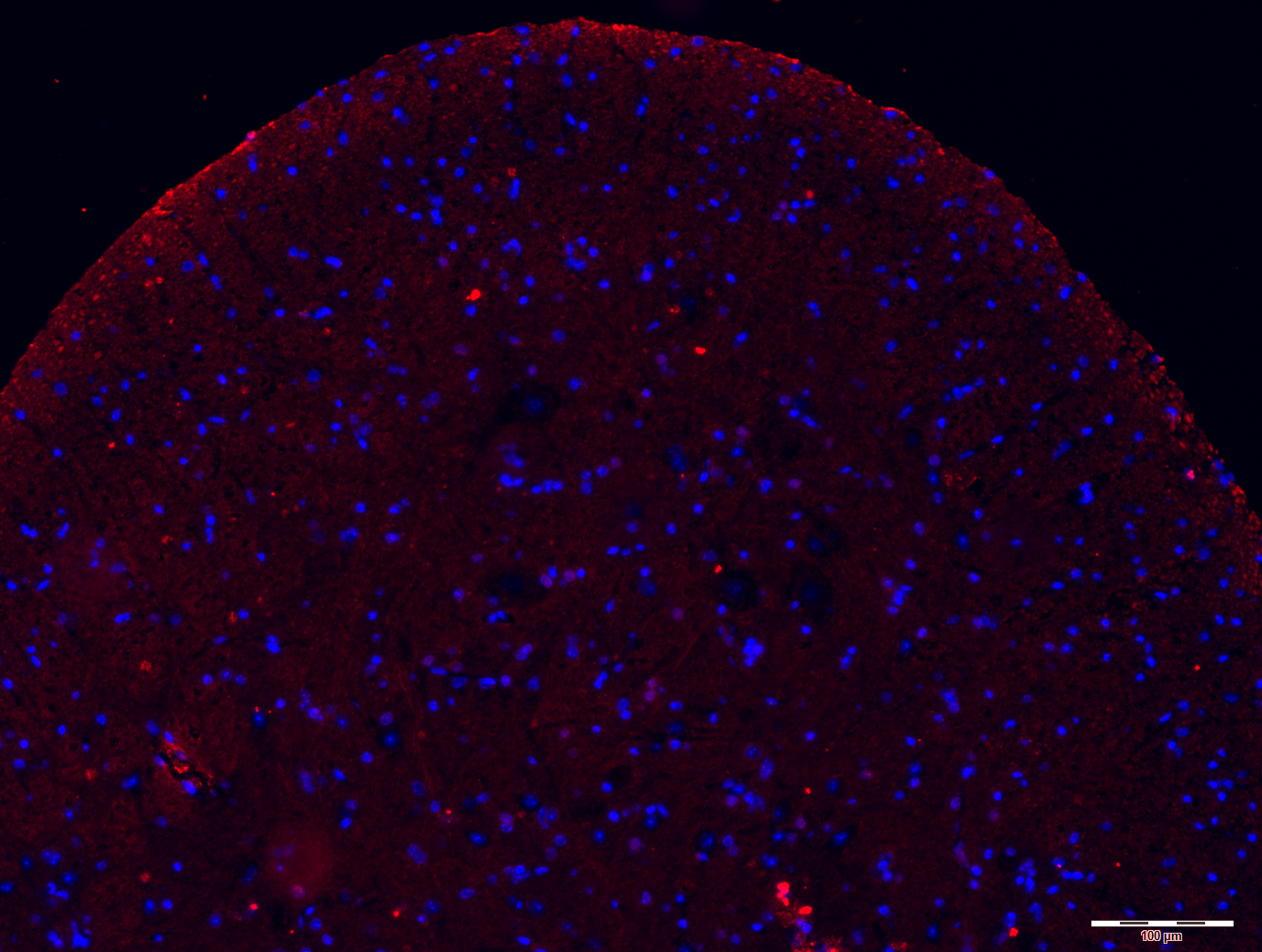

Supplement: S1 File — (ZIP) [file pone.0292584.s001.zip › pone0170825 files/Figure 9/RANTES_EAE+fingo_corno ant_10x.tif]

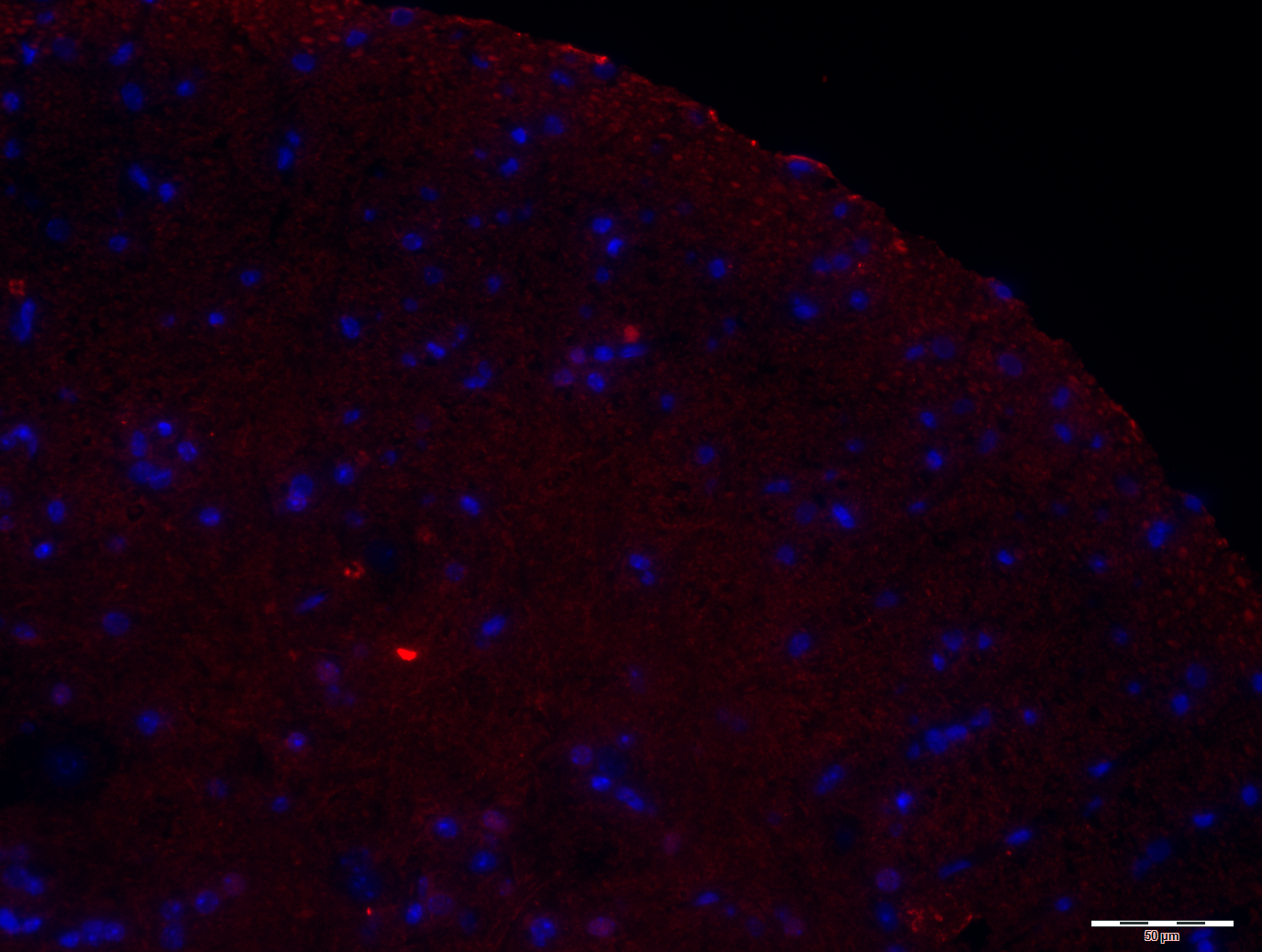

Supplement: S1 File — (ZIP) [file pone.0292584.s001.zip › pone0170825 files/Figure 9/RANTES_EAE+fingo_corno ant_20x.tif]
